# Characterization of Ionizable Lipid Membranes for Designing Bioactive Interface of Inorganic Particles

**DOI:** 10.1021/acs.langmuir.6c04968

**Published:** 2026-09-23

**Authors:** Taisei Suzuki, Keishi Suga, Ikumi Kato, Takeru Yamazaki, Kanako Watanabe, Tom A. J. Welling, Hikaru Namigata, Noriko Yamauchi, Kumiko Hayashi, Young-Tae Chang, Satoshi Arai, Daisuke Nagao

**Affiliations:** † Department of Chemical Engineering, Tohoku University, 6-6-07, Aoba, Aramaki-Aza, Aoba-Ku, Sendai 9808579, Japan; ‡ Nano Life Science Institute (WPI-NanoLSI), Kanazawa University, Kakuma-Machi, Kanazawa 9201192, Japan; § Frontier Research Institute for Interdisciplinary Sciences, Tohoku University, 6-3, Aoba, Aramaki-Aza, Aoba-Ku, Sendai 9808579, Japan; ∥ Domain of Materials Science and Engineering, Faculty of Applied Science and Engineering, Ibaraki University, 4-12-1, Nakanarusawa-Cho, Hitachi 316-8511, Japan; ⊥ The Institute for Solid State Physics, The University of Tokyo, 5-1-5 Kashiwano-Ha, Kashiwa 277-8581, Japan; # Department of Chemistry, 34995 Pohang University of Science and Technology, Pohang 37673, Republic of Korea

## Abstract

Magnetic nanoparticles and their composites are attractive candidates for novel photothermal therapy (PTT) agents in cancer treatment. However, the cellular uptake of these artificial particles remains insufficient because foreign materials are easily excreted from cells. Although lipid membrane coating offers a potential solution to this issue, the specific roles of lipid membrane properties, such as membrane fluidity, polarity, and pH responsive surface charge, remain unclear. Herein, we investigated the physicochemical properties of lipid bilayers containing the ionizable cationic lipid, SM-102, and utilized them to functionalize photothermal magnetic composite particles (MCPs). Liposome characterization revealed that the incorporation of SM-102 slightly decreased the membrane fluidity. Coating the photothermal MCPs with SM-102 bilayers successfully imparted pH responsiveness. Consequently, these lipid-coated MCPs (L-MCPs) exhibited enhanced cellular uptake of particles in HeLa cells at acidic pH. Although both uncoated MCPs and L-MCPs were nontoxic under normal conditions, they exhibited cytotoxicity upon simultaneous exposure to NIR (808 nm) light and a direct current magnetic field (150 mT). Local temperature analyses revealed that L-MCP internalization led to elevated local temperatures in the mitochondria, whereas local heating in the lysosomes was reduced compared to pristine MCPs, indicating that coating with SM-102-containing lipid membranes can alter intracellular particle positioning.

## Graphical abstract

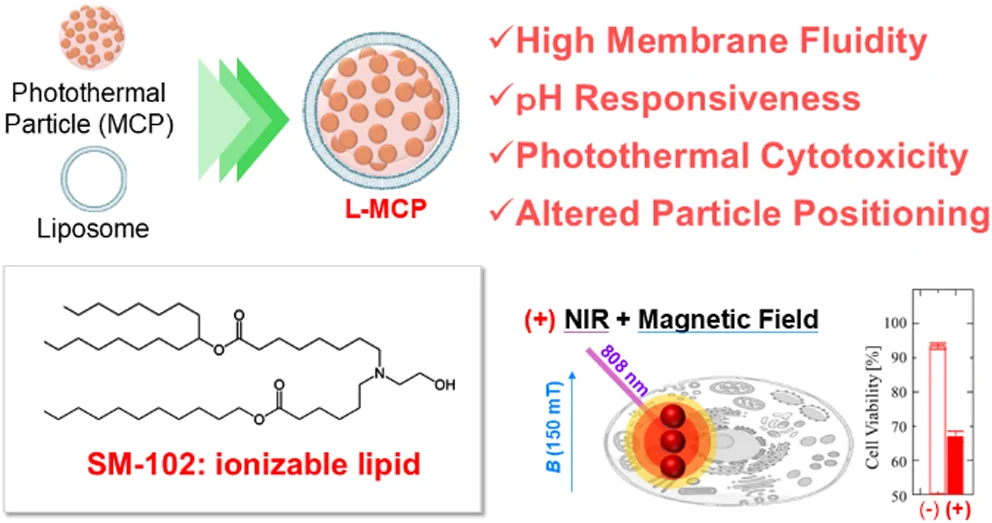

## 1 Introduction

Nanoparticles are widely utilized in clinical cancer treatments, including chemotherapy, radiation therapy, targeted therapy, and immunotherapy. Recently, various photothermal materials have been developed to realize efficient photothermal therapy (PTT).^1–6^ In PTT, agents capable of converting light into heat are delivered to target cells, which are then thermally ablated upon near-infrared (NIR) laser irradiation. Iron oxide nanoparticles act not only as photothermal heat mediators^7,8^ but also as catalyst precursors for ferroptosis.^9–11^ However, the in vivo application of inorganic materials is often limited by their potential toxicity. Although silica (SiO_2_) is biocompatible, its size and architecture must be carefully designed in terms of biosafety.^12–15^ Furthermore, nanoparticle aggregation drastically reduces their surface functions; therefore, ensuring colloidal stability in physiological environments is essential. To overcome these challenges, surface functionalization with organic materials, such as polymers and lipid membranes, is currently being investigated.^7,16–19^

Magnetic nanoparticles (MNPs), such as Fe_3_O_4_, and their composites are versatile intracellular heating agents.^1,20^ We previously developed magnetic composite particles (MCPs), composed of MNPs and support materials, which exhibit unique characteristics such as superparamagnetism, photothermal conversion ability, and biocompatibility.^7^ To optimize the photothermal cytotoxicity of MCPs, their dispersibility and cellular uptake efficiency must be improved. Generally, an increase in hydrodynamic particle size, especially beyond 500 nm, significantly decreases cellular uptake.^21,22^ Consequently, for clinical applications, the particle size should be controlled within the range of 50–300 nm to facilitate efficient cellular internalization upon contact with target cell membranes.

Surface modification of nanoparticles plays a crucial role both prior to and following endocytosis. Because endocytic pathways are initiated by physical contact with the negatively charged plasma membrane, a cationic nanoparticle surface is highly effective in enhancing cellular uptake. After endocytic internalization, particles are typically degraded or excreted unless they escape from endosomes or lysosomes.^23,24^ Therefore, innovative strategies are required to enhance particle uptake and to control intracellular localization. To this end, ionizable cationic lipids are widely utilized in drug delivery, especially for mRNA therapeutics.^25–28^ In addition, the dynamic properties of lipid nanoparticles (or liposomes) must be considered: a high-fluidity lipid membrane has been reported to promote membrane fusion, thereby enhancing drug delivery efficacy.^29–31^

Coating inorganic nanoparticles with lipids or cell-derived membranes is an effective strategy to functionalize their surfaces.^32–34^ Herein, we employed the ionizable cationic lipid, SM-102 (heptadecan-9-yl-8-((2-hydroxyethyl)­(6-oxo-6-(undecyloxy)­hexyl)­amino)­octanoate) (Figure 1a). SM-102 is a pH-responsive lipid (p*K*_a_ ≈ 6.75)^25^ widely employed in the mRNA vaccine Spikevax.^35^ Its branched tail structure is also expected to facilitate endosomal escape.^36^ However, the physicochemical properties of such lipid membranes on inorganic nanoparticles have not yet been sufficiently investigated. This study aims to functionalize photothermal composite particles (MCPs) via lipid membrane coating to enhance intracellular particle delivery. First, we systematically investigated the effects of SM-102 on lipid bilayer properties (fluidity, polarity, and pH responsiveness) using liposomes. It was then confirmed that the membrane properties of SM-102 liposomes were preserved even when decorated onto SiO_2_ particles, suggesting that lipid molecules on inorganic particle surfaces behave similarly to these in free-suspending liposomes. Next, we fabricated silica-based MCPs (SiO_2_@MNP@SiO_2_) with a total diameter of approximately 200 nm and coated them with SM-102 lipid bilayers. The dispersibility and surface charge of these lipid-coated MCPs (L-MCPs) were characterized. Finally, we evaluated the cellular uptake and cytotoxicity of the particles in HeLa cells with/without NIR irradiation, and discussed the effect of the lipid coating by comparing the local heating efficacy within specific organelles, including the endoplasmic reticulum (ER), lysosomes, mitochondria, and plasma membrane.

**Figure 1:**
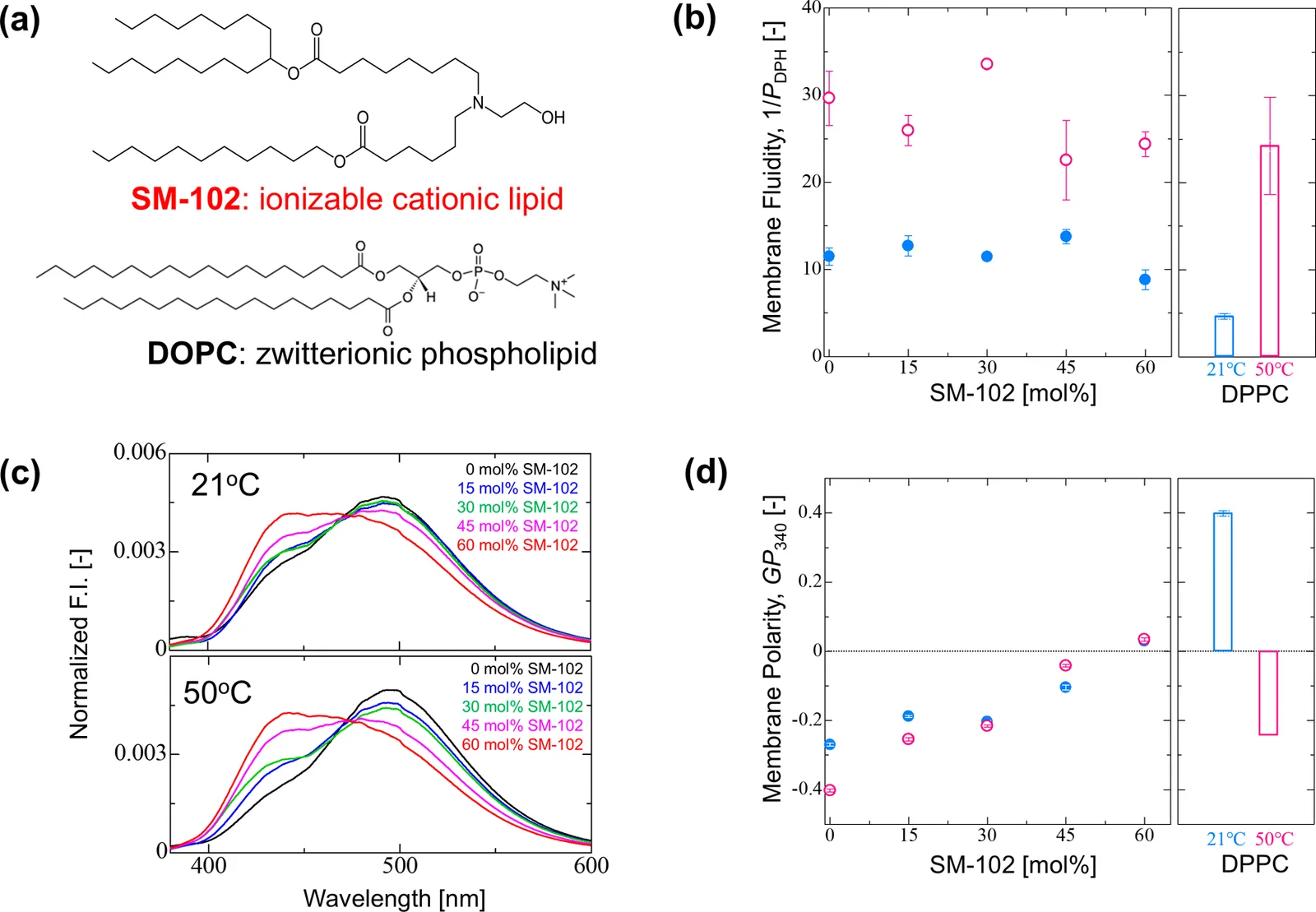
(a) Chemical structures of the lipids. (b) Membrane fluidity (1/*P*_DPH_) of SM-102/DOPC liposomes at various molar compositions, measured at 21 °C (filled symbols, cyan) and at 50 °C (open symbols, magenta). (c) Steady-state fluorescence emission spectra of Laurdan in SM-102/DOPC liposomes. (d) Membrane polarity (GP_340_) of SM-102/DOPC liposomes at various molar compositions, measured at 21 °C (filled symbols, cyan) and at 50 °C (open symbols, magenta).

## 2 Materials and Methods

### 2.1 Materials

MCPs, composed of a core particle, magnetic nanoparticles (Fe_3_O_4_) and silica, were fabricated based on our previous reports.^7,37,38^ Details including material provider information are shown in Supporting Information. Liposomes were composed of SM-102 (heptadecan-9-yl-8-((2-hydroxyethyl)­(6-oxo-6-(undecyloxy)­hexyl)­amino)­octanoate) and DOPC (1,2-dioleoyl-*sn*-glycero-3-phosphocholine) at different molar ratios. Fluorescent thermometers (Organelle Thermo Greens (OTGs)) were used to measure local temperatures.^39,40^ Water was deionized in advance (>18.2 MΩ cm).

### 2.2 Characterization of Lipid Membrane Properties

Particle size distributions and zeta potentials of the particles in aqueous medium were evaluated by dynamic light scattering (DLS; ELSZ-2 (Otsuka Electronics)). Membrane fluidities (1/*P*_DPH_) of liposomes and lipid-coated SiO_2_ (L-SiO_2_) were evaluated based on a steady-state fluorescent anisotropy measurement (Supporting Information; eqs S4 and S5).^41,42^ Surface charge density of particles was evaluated by a fluorescent probe 6-(*p*-toluidino)-2-naphthalenesulfonic acid sodium salt (TNS) (Supporting Information; eqs S6–S9).^43–45^ Membrane polarities (GP_340_) were evaluated based on a steady-state fluorescent emission spectrum of Laurdan (Supporting Information; eq S10).^41,46,47^ The thickness of the lipid bilayer was evaluated by DPH fluorescence quenching method (Supporting Information; eq S13).^41,48^

### 2.3 Preparation of Lipid Coated Particles

The lipid-coated SiO_2_ (L-SiO_2_) and lipid-coated MCPs (L-MCPs) were prepared by a modified coextrusion method.^49^ The fabrication procedure is shown in Figure S1a. Herein, the ratio of total liposome surface area (*A*_liposome_) to total core particle surface area (*A*_particle_) was 20:1 to achieve sufficient surface coverage (Supporting Information; Figure S1b,c). The suspensions of particles (SiO_2_ or MCPs) and liposomes were mixed and heated to 60 °C. The suspensions were extruded 11 times through a polycarbonate membrane with a mean pore diameter of 400 nm, using an extruding device (Liposofast; Avestin Inc., Ottawa, ON, Canada). The suspensions obtained were washed by centrifugation (2300*g* for 5 min with deionized water), and redispersed in deionized water. The obtained particles were characterized by a transmission electron microscope (TEM, Hitachi, HD-2700), a dynamic light scattering (DLS, Otsuka Electronics, ELSZ-2), and a vibrating sample magnetometer (VSM, Toei Scientific Industrial Co., PV-M20–5). L-SiO_2_ was used for characterizing membrane properties. L-MCPs was used to evaluate cellular uptake, photothermal conversion and cytotoxicity, and local temperature measurements.

### 2.4 Cell Culture

HeLa cells were obtained from the JCRB Cell Bank (National Institutes of Biomedical Innovation, Health, and Nutrition, Japan). The cells were seeded into a glass-bottom dishes (ϕ35 mm, D11130H; Matsunami Glass Ind, Ltd.) and maintained in DMEM (Thermo Fisher Scientific) supplemented with 10% fetal bovine serum (Thermo Fisher Scientific) and 1% penicillin-streptomycin (Thermo Fisher Scientific) at 37 °C in a humidified atmosphere of 5% CO_2_.

To express GFP (Green Fluorescence Protein) in the cytoplasm of Hela cells, pEGFP-C1 plasmid DNA (Clontech Laboratories, Inc.) was transfected into the cells using Lipofectamine 3000 (Thermo Fisher Scientific) according to the manufacturer’s protocol. The GFP-expressing HeLa cells were cultured in DMEM containing MCPs or L-MCPs (final particle concentration: 200 μg/mL) at 37 °C for 24 h in a humidified atmosphere of 5% CO_2_. The pH of the culture medium was adjusted to 7.4 or 6.7 using a buffer composed of citric acid, disodium hydrogen phosphate and HEPES. The cells were washed three times with HBSS (Thermo Fisher Scientific) and subsequently observed using a confocal laser scanning microscope (CLSM, Nikon, C2–Si), equipped with a 100× oil immersion objective lens in the FITC (emission filter set: 490–540 nm; excitation laser: 488 nm) for GFP and the TRITC (emission filter set: 575–675 nm; excitation laser: 561 nm) for the particles. The CLSM images were acquired at a resolution of 1024 × 1024 pixels (0.12 μm/pixel) and analyzed as 16-bit intensity images using ImageJ/Fiji software.

### 2.5 Cell Viability Test

Live/dead cell viability assay was performed using calcein AM (PromoCell GmbH, Germany) and ethidium homodimer III (EthD-III) (PromoCell GmbH, Germany), based on the previously reported method.^50^ HeLa cells were cultured with MCPs or L-MCPs for 24 h. After a NIR laser irradiation (10 min, λ = 808 nm, 5.0 W/cm^2^) under a direct current magnetic field (DCMF, *B* = 150 mT), the cells were stained by calcein AM and EthD-III. Green fluorescence and red fluorescence indicated live cells and dead cells, respectively. At least 500 cells per dish were analyzed, and the cell viability is calculated as follows

1 $$c\mathrm{ell}\;\mathrm{viability}\;\left[\%\right]=\frac{N_{green}}{N_{green}+N_{red}}\times 100$$

where *N*_green_ and *N*_red_ indicate the numbers of Calcein AM-stained cell and EthD-III-stained cell, respectively.

### 2.6 Local Temperature Measurements

Four types of OTGs,^39,40^ including ER Thermo Green (ETG), Lysosome Thermo Green (LTG), Mitochondria Thermo Green (MTG), and Plasma membrane Thermo Green (PTG), were utilized to evaluate localized photothermal behavior. Herein, the relative change in fluorescence intensity before and during NIR irradiation was analyzed to estimate the temperature increase (Δ*T*) at specific organelles. HeLa cells were cultured with MCPs or L-MCPs (final particle concentration: 200 μg/mL) at 37 °C for 24 h in a humidified atmosphere of 5% CO_2_. The cells were washed three times with HBSS, then stained with one of the OTGs at a final concentration of 1 μM. After 30 min incubation at 37 °C, the cells were observed by a CLSM (FV1200, Olympus, Tokyo, Japan), equipped with a 60× oil immersion objective lens (NA = 1.42, PLAPON, Olympus, Tokyo, Japan) in the FITC channel (emission filter set: 490–540 nm; excitation laser: 473 nm) and Texas Red channel (emission filter set: 575–675 nm; excitation laser: 561 nm) for OTGs and MCPs (or L-MCPs), respectively. An NIR laser (808 nm, 6 mW, spot diameter of 10 μm, LuxX 808–190, Omicron, Dudenhofen, HE, Germany) was applied for 10 s. To confirm the reversibility of thermal quenching, NIR laser illumination was repeated three times with 10-s intervals.

## 3 Results and Discussion

### 3.1 Physicochemical Properties of SM-102 Incorporated Liposomes

The physicochemical properties of liposome membranes, including membrane fluidity (quantified as 1/*P*_DPH_ using DPH), membrane polarity (expressed as GP_340_ using Laurdan), and pH responsive surface charge, are key factors regulating cellular uptake.^30,51–53^ The roles of SM-102 in DOPC liposomes were investigated (Figure 1). Although the membrane fluidity (1/*P*) slightly decreased at 60 mol % SM-102 (Figure 1b) at 21 °C, these fluidity values were distinctly higher than those of a solid-ordered (*S*_o_) phase membrane (i.e., DPPC at 21 °C, which is below its phase transition temperature (*T*_m_) of approximately 42 °C). The membrane fluidity increased upon heating, confirming the liquid-disordered (*L*_d_) phase of the SM-102/DOPC liposomes. Among cationic lipids, DOTAP (1,2-dioleoyl-3-trimethylammonium propane, C18:1) is reported to increase membrane fluidity due to its unsaturated hydrocarbon tails,^54^ whereas DPTAP (1,2-dipalmitoyl-3-trimethylammonium propane, C16:0) and DODAB (dimethyldioctadecylammonium bromide, C18:0) decrease membrane fluidity due to their saturated hydrocarbon tails.^54,55^ Although SM-102 has three saturated tails, its fluidity decreasing effect was minor, likely owing to its relatively short hydrocarbon chains (C8:0, C8:0, C12:0).

The micropolarity of membranes was investigated using a solvatochromic dye Laurdan. The steady-state emission spectrum of DOPC exhibited a unimodal peak at approximately 490 nm, confirming the *L*_d_ phase. With an increasing SM-102 molar ratio, the emission spectra became bimodal (Figure 1c). The peak intensity at approximately 440 nm (corresponding to the ordered phase) increased with the SM-102 ratio, suggesting that SM-102 induced membrane dehydration. The membrane polarity (GP_340_) is a complementary parameter to membrane fluidity: a negative value (GP_340_ < 0) indicates a hydrated interface (e.g., the *L*_d_ phase), whereas a positive value (GP_340_ > 0) indicates a dehydrated interface (e.g., the *S*_o_ phase or the liquid-ordered (*L*_o_) phase induced by the presence of cholesterol or its derivatives).^41–43,46,54,56^ The GP_340_ value increased in proportion to the molar ratio of SM-102 and became positive at 60 mol % SM-102. In addition, no significant temperature dependency of GP_340_ was observed in the 60 mol % SM-102 liposomes. Such behavior is characteristics of cholesterol-incorporated liposomes.^41^ Considering that SM-102 possesses a hydroxy group, a hydrogen bonding interactions between SM-102 and DOPC are expected, which could induce weak dehydration and subsequently lead to the observed increase in GP_340_.

The zeta potentials (ζ) of the liposomes were investigated (Figure S2a); SM-102/DOPC liposomes exhibited zeta potential values greater than 30 mV at pH 3.4, whereas those values dropped below 5 mV at pH 9.3. Because the p*K*_a_ value of SM-102 is 6.75,^25^ this lipid successfully imparts pH responsiveness to the liposomes. The ζ values reached a plateau at SM-102 ratios exceeding 15 mol %. On the other hand, the adsorption capacity of the anionic dye TNS, which quantitatively indicates the number of cationic groups at the interface,^44,45^ increased in a dose-dependent manner in proportion to the SM-102 molar ratio (Figure S2b). Notably, the number of cationic groups at the liposome surface reached a plateau at an SM-102 ratio of 30 mol %.

High membrane fluidity and a pH-dependent surface charge are critical lipid bilayer properties required to achieve cancer cell-specific interactions.^26,29^ By comparing liposomes with various molar ratios of SM-102 to DOPC, we determined that the optimal composition was an SM-102/DOPC ratio of 3:7. This specific formulation provides both high membrane fluidity and a hydrated surface (GP_340_ < 0), while successfully maintaining pH responsiveness.

### 3.2 Functionalities of Lipid Coated Particles

SM-102/DOPC (3:7 molar ratio) liposomes were coated onto inorganic particles via a coextrusion method.^49^ SiO_2_ (volume-average diameter, *d*_V_ = 199 nm) and MCP (*d*_V_ = 229 nm) were used for lipid coating (see Figure S3 for details). Figure 2 shows a TEM image of the MCPs after lipid coating. The magnetic nanoparticles were decorated onto the SiO_2_ core particle, and the outermost layer was coated with a shell of relatively low contrast. Herein, the lipid-coated particles (L-SiO_2_ and L-MCP) were characterized to confirm the functional properties derived from the coated layer.

**Figure 2:**
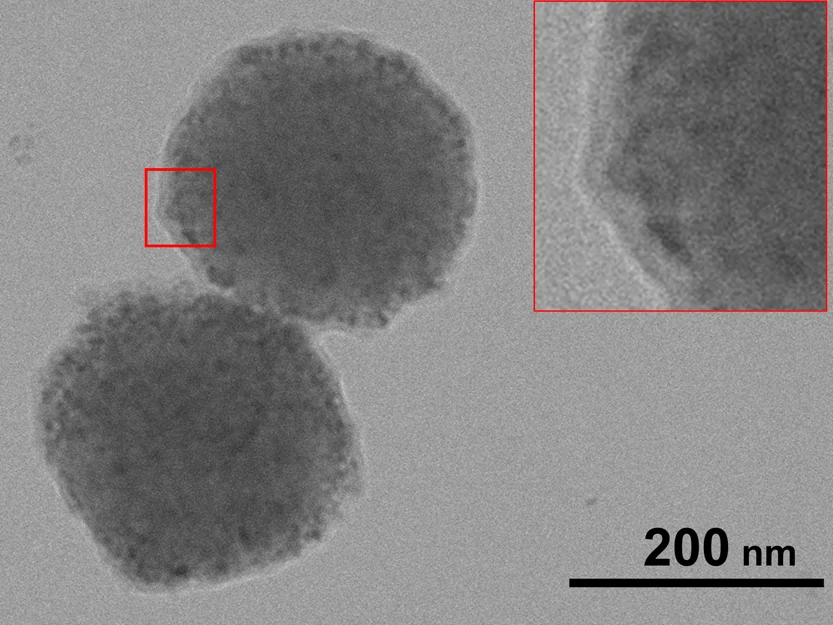
TEM image of the L-MCPs (inset: magnified view showing the surface layer).

#### 3.2.1 Membrane Fluidity

SiO_2_ was used as a model substate for the membrane fluidity study to avoid the intrinsic fluorescence of the MCPs (i.e., RBITC) during DPH measurements. The membrane fluidity was compared between the liposomes and lipid-coated SiO_2_ (L-SiO_2_) (Figure 3a). At 21 °C, similar 1/*P* values were obtained for the SM-102/DOPC liposomes and L-SiO_2_, and these values were significantly higher than that of DPPC (1/*P* = 4.6 ± 0.6 at 21 °C, *S*_o_ phase). This demonstrates that the coated membranes maintain high fluidity. Both the SM-102/DOPC liposomes and L-SiO_2_ exhibited a temperature-dependent increase in membrane fluidity; however, the 1/*P* values of L-SiO_2_ were slightly smaller than those of the corresponding liposomes. This difference could be attributed to the interaction between the solid support surface and the lipid polar headgroups, which restricts the lateral diffusion of lipids.^57^

**Figure 3:**
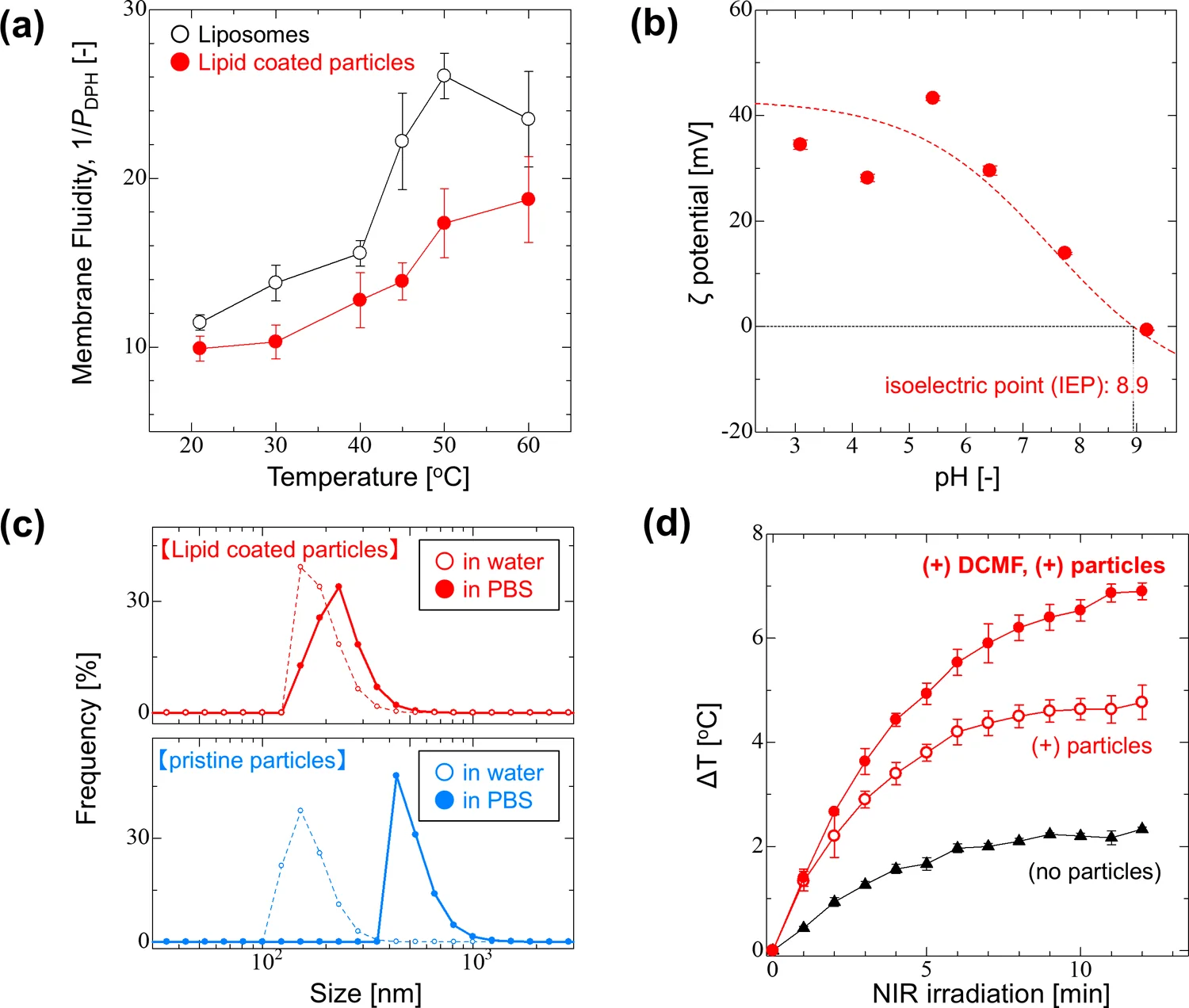
(a) Membrane fluidity (1/*P*_DPH_) of the L-SiO_2_ particles (filled symbols) and free suspending liposomes (SM-102/DOPC (3:7)). (b) pH-dependent ζ potentials of the L-MCPs. The dotted line indicates the fitting curve based on the Henderson–Hasselbalch equation. (c) Particle size distributions of L-MCPs (top) and pristine MCPs (bottom), measured in water and in PBS. (d) Photothermal performance of the L-MCPs under NIR irradiation (λ = 808 nm, 5 W/cm^2^) without or with a DCMF (*B* = 150 mT). Symbols indicate the control (PBS only; triangles), L-MCP with NIR (open circles), and L-MCP with combined NIR and DCMF (filled circles).

#### 3.2.2 pH-Dependent Surface Charge

The zeta potential (ζ) of pristine MCPs in water was −28 mV, indicating that their surface properties are essentially identical to those of silica. After coating with the SM-102/DOPC (3:7) liposomes, the resulting L-MCPs exhibited tunable zeta potentials; specifically, the ζ values exceed +30 mV at pH conditions below 6 (Figure 3b). In addition, the ζ values became negative (ζ < 0) at pH conditions above 8.0, which is attributed to the zwitterionic nature of DOPC. Because the surface of pristine MCPs consists primarily of silica,^37,38^ their isoelectric point (IEP) is expected to be in the pH range of 2–3. In contrast, the IEP of the L-MCPs was estimated to be 8.9, which is comparable to that of free suspending SM-102/DOPC (3:7) liposomes (Figure S4). These results demonstrate the successful decoration of the SM-102-incorporated lipid membrane onto MCPs.

The L-MCPs exhibited good dispersibility in PBS (pH 7.4), whereas the dispersibility of pristine MCPs in PBS was poor (Figure 3c). The hydrodynamic diameters (*D*_10_, *D*_50_, *D*_90_), span value (= (*D*_90_–*D*_10_)/*D*_50_), polydispersity indices (PDI), and hydrodynamic diameter (*D*_h_) are summarized in Table 1. No significant differences in span values were observed between pristine MCPs and L-MCPs, whether in water or in PBS, indicating that the width of the particle size distribution was comparable after lipid coating. Common to both MCPs and L-MCPs, the values of PDI in PBS were higher than those in water, suggesting that both particle types undergo partial aggregation in PBS. However, comparing the hydrodynamic diameter (*D*_h_), L-MCPs in PBS exhibited smaller *D*_h_ values than pristine MCPs. These results demonstrate that the lipid coating effectively suppresses particle aggregation under high ionic strength conditions. Unmodified inorganic particles generally exhibit poor dispersibility in the aqueous media with high ionic strength.^7^ At the physiological pH of PBS (7.4), the surface charge of L-MCPs was not highly pronounced, suggesting minimal electrostatic repulsion between the particles. However, the lipid bilayer shell on the particle provides steric stabilization. This is because the surface of *L*_d_ phase lipid membrane is highly hydrated,^47,58^ thereby preventing aggregation in high ionic strength media. Consequently, the lipid coating allowed the L-MCPs to maintain particle sizes appropriate for endocytic internalization.^21^

**Table 1:** Hydrodynamic Diameters and Polydispersity Indices (PDIs) of the Particles

	medium	*D*_10_ [nm]	*D*_50_ [nm]	*D*_90_ [nm]	PDI [-]	span [-]^a^	*D*_h_ [nm]^b^
MCP	water	114 ± 11	144 ± 10	200 ± 13	0.058	0.599	245 ± 6
	PBS	367 ± 2	440 ± 13	628 ± 15	0.307	0.593	1256 ± 197
L-MCP	water	130 ± 1	162 ± 2	227 ± 2	0.081	0.596	305 ± 5
	PBS	162 ± 27	197 ± 27	278 ± 37	0.276	0.590	521 ± 24

a Calculated by (*D*_90_–*D*_10_)/*D*_50_.

b Hydrodynamic diameter calculated by cumulant method.

#### 3.2.3 Photothermal Conversion and Magnetic Assembly

A potential concern arises that lipid bilayers might restrict the original function of MCPs, such as photothermal conversion and chain-like assembly under a direct current magnetic field (DCMF). However, L-MCPs exhibited a photothermal conversion ability (Figure 3d) comparable to that of pristine MCPs (Supporting Information; Figure S5). Specifically, 10 min of NIR irradiation (808 nm, 5 W/cm^2^, dispersion volume of 3 mL, and particle concentration of 200 μg/mL) resulted in a temperature rise of 4 °C in the dispersion. By applying a DCMF (*B* = 150 mT), the photothermal conversion efficiency increased 1.5-fold, resulting in a 6 °C increase in temperature. The magnetic assembly and redispersal of the L-MCPs was confirmed via optical microscopy (Figure S6). These results demonstrate that the lipid membrane coating did not impede the photothermal conversion ability of the MCPs under a DCMF.

The physicochemical and magnetic properties of MCPs depend on the amount of incorporated magnetic nanoparticles (Table S1). The mass fraction of the MNPs was found to correlate directly with the specific surface area of the SiO_2_ core, suggesting monolayer coating of the MNPs (Figure S7a). The photothermal conversion efficiency per unit mass (Δ*T*/*W*_MCPs_) depended directly on the mass fraction of the incorporated MNPs (Figure S7b). In summary, superior efficient photothermal conversion can be rationally achieved using MCPs with high MNP loading, while bigger MCPs are advantageous in terms of magnetic assembly.^7^

The photothermal conversion efficiency of Fe_3_O_4_ nanoparticles is highly sensitive to their state of aggregation;^1,3^ specifically, the efficiency increases as the interparticle distance decreases. The magnetic assembly of MCPs shortens the distance between the constituent magnetic particles within the composite, thereby enhancing their photothermal conversion capability.^3,59^ Although steric stabilization is often considered a factor that for decreasing photothermal conversion efficiency, our lipid coating did not hinder photothermal conversion, likely because the thickness of the lipid bilayer is usually less than 5 nm (50 Å). The membrane thickness of SM-102/DOPC (3:7 molar ratio) bilayer was investigated (Supporting Information; Table S2; Figure S8). Based on the fluorescence quenching method,^48^ the membrane thickness of the SM-102/DOPC (3:7 molar ratio) liposomes was 31.0 ± 0.5 Å, which is comparable to that of pure DOPC liposomes (32.0 ± 0.4 Å). This result suggests that SM-102 lipids were fully miscible with DOPC bilayer, as illustrated in Figure S8. Following coating, the membrane thickness of L-SiO_2_ was 33.2 ± 0.6 Å, confirming unilamellar lipid bilayer coating. Consequently, lipid coating successfully improved the dispersibility of MCPs in PBS while preserving their magnetic assembly under a DCMF and their photothermal conversion abilities. Furthermore, the surface charge of L-MCP becomes positive under acidic conditions, which is expected to enhance their cellular uptake.^60^

### 3.3 Cellular Uptake and Cytotoxicity

#### 3.3.1 Cellular Uptake

The intracellular internalization behavior of the particles was investigated in HeLa cells. MCPs or L-MCPs were incubated with HeLa cells (at total particle concentration of 200 μg/mL) at pH 6.7 or 7.4. After 24 h, the particle uptake was evaluated based on the luminance observed via CLSM. Without the lipid coating, the pH of the medium did not affect the uptake amount (Figure 4a). Because the surface of pristine MCPs consists primarily of silica (with an isoelectric point (IEP) of pH 2–3),^37,38^ their surface charge is expected to remain unchanged during the endocytosis process. In contrast, the internalization of L-MCPs significantly increased at pH 6.7 (Figure 4b). The IEP of L-MCPs was 8.9 (Figure 3b), which corroborates that these particles possess a strong positive surface charge under acidic conditions.

**Figure 4:**
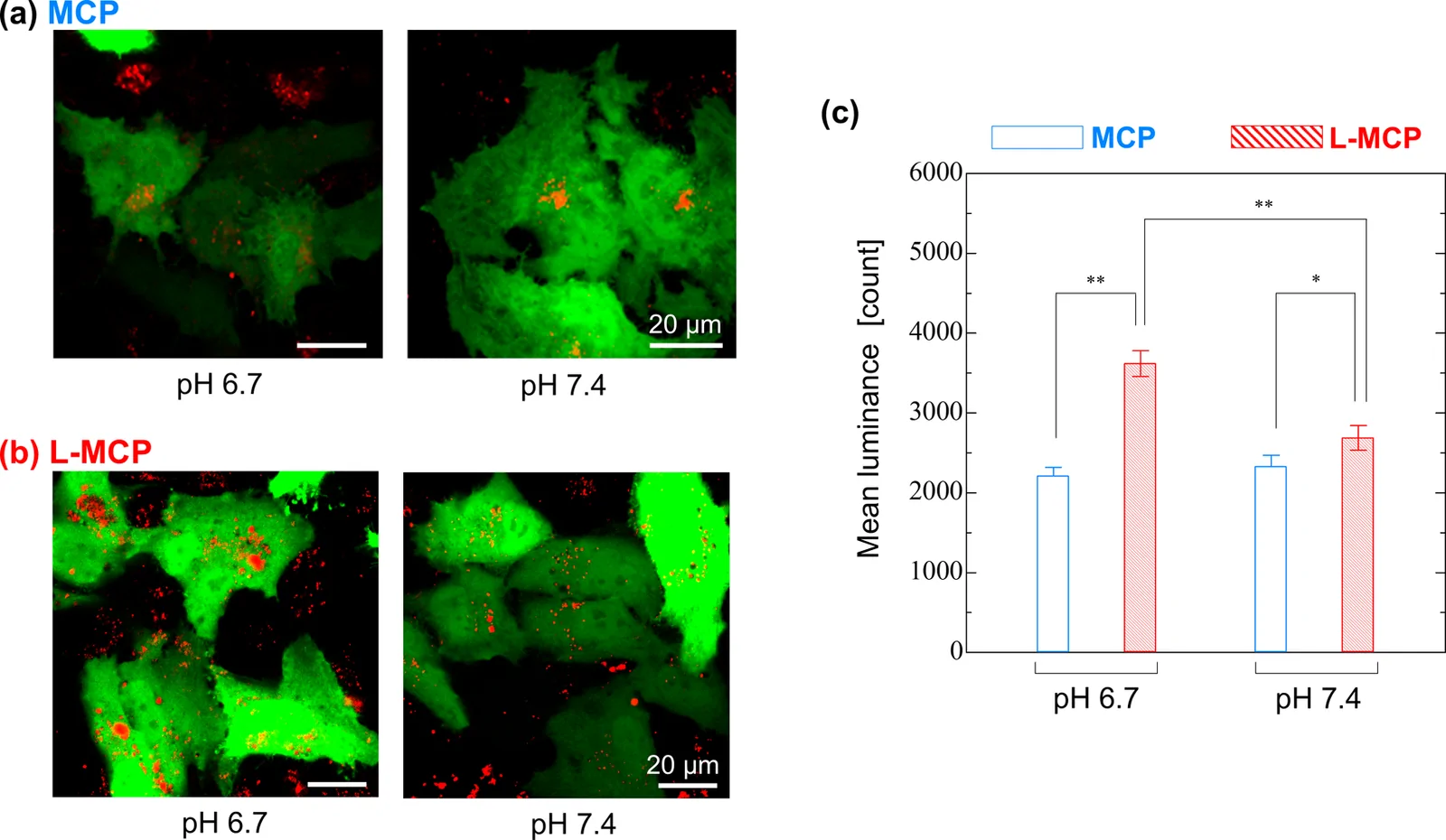
CLSM images of HeLa cells treated with (a) pristine MCPs or (b) L-MCPs at pH 6.7 or 7.4. Scale bar: 20 μm. Red and green fluorescence correspond to the particles and GFP (cytoplasm), respectively. The applied particle concentration was 200 μg/mL. (c) Cellular uptake of the particles, evaluated based on the mean luminance values observed inside the cells. Histograms of the luminance are shown in Supporting Information (Figure S9). Statistical significance was determined by Student’s *t*-test (*p < 0.05 and **p < 0.01).

The distributions of the luminance from internalized particles were evaluated (Supporting Information; Figure S9), and the mean luminance values were compared (Figure 4c). The mean luminance for L-MCPs at pH 6.7 was the highest among the four conditions, demonstrating the efficient particle uptake of the SM-102 modified particles under a weakly acidic condition. At pH 6.7, the surface of the L-MCPs bears a positive charge (Figure 3b), which promotes initial physical contact with the plasma membrane via electrostatic interactions.^61^ Ionizable cationic lipids, such as SM-102, play a crucial role in the internalization of lipid nanoparticles.^62^ Notably, the difference in the uptake amounts between MCPs and L-MCPs at pH 7.4 was statistically significant but marginal (*p* < 0.05).

#### 3.3.2 Cytotoxicity

A live/dead assay was performed for pristine MCPs and L-MCPs. No apparent cytotoxicity was observed for HeLa cells treated with either MCPs or L-MCPs at applied particle concentrations up to 1000 μg/mL (Figure 5a). At pH 7.4, the incorporated SM-102 is only partially ionized (not fully ionized), which likely mitigates the potential cytotoxicity typically associated with cationic surface. In contrast, MCPs functionalized with a polyethylenimine (PEI),^7^ or with a 1,2-dioleoyl-3-trimethylammonium-propane chloride (DOTAP) lipid (Figure S7), exhibited pronounced cytotoxicity. At the liposome level, lipids with quaternary ammonium headgroup exhibited higher cytotoxicity than those with tertiary amine headgroup.^63^ Furthermore, particle surfaces covered with quaternary ammonium groups caused significant damage to model lipid membranes.^44^ Consequently, coating cargo particles with ionizable cationic lipids is beneficial for mitigate cytotoxicity.

**Figure 5:**
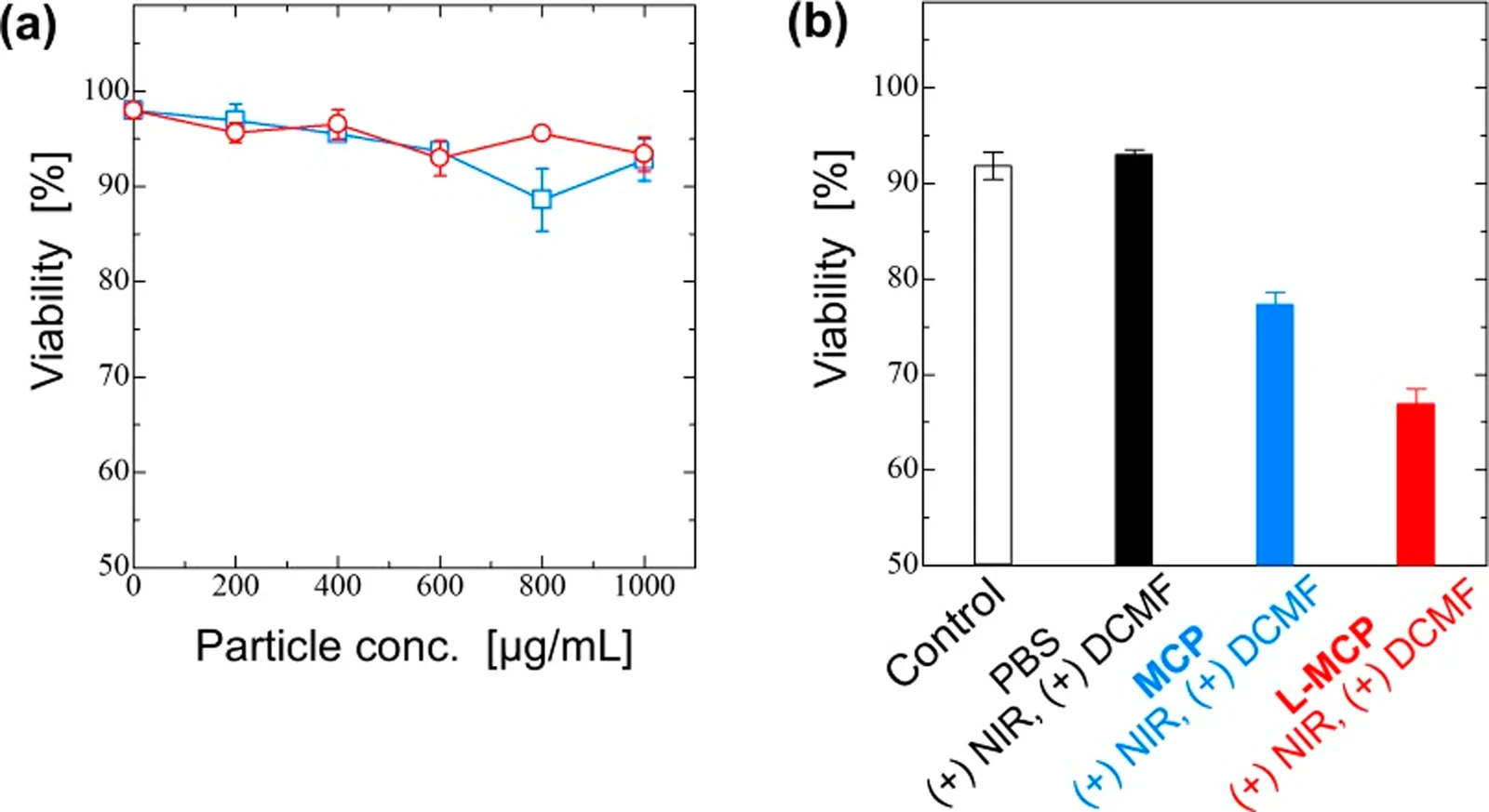
Cytotoxicity of the particles toward HeLa cells. (a) Cell viability in the presence of pristine MCPs (square) and L-MCPs (circle). (b) Cell viability after a 10 min treatment with combined NIR irradiation (λ = 808 nm, 5 W/cm^2^) and a DCMF (*B* = 150 mT). “Control” indicates samples evaluated in the absence of particles, NIR irradiation, and the DCMF.

We previously reported the effect of NIR irradiation alone;^7^ in the absence of particles, cell viability remained high (approximately 94%) following 10 min NIR irradiation, confirming that a 10 min NIR irradiation is harmless to HeLa cells. Furthermore. Wang et al. demonstrated that exposure to a static magnetic field (i.e., DCMF) alone did not affect the viability of HeLa cells.^64^ Taken together with our control experiments, the combined application of NIR irradiation and a DCMF causes no synergistic cytotoxic effects in the absence of MCPs.

Importantly, NIR irradiation combined with the application of a DCMF (*B* = 150 mT) successfully decreased the viability of HeLa cells in the presence of particles (200 μg/mL) (Figure 5b). Specifically, applying both NIR and the DCMF to cells treated with pristine MCPs decreased cell viability from 90% to 77%. For the L-MCPs, this combined treatment resulted in a more pronounced decrease in viability, from 93% to 67%. Despite comparable cellular uptake at pH 7.4, the L-MCPs exhibited significantly higher photothermal cytotoxicity than the pristine MCPs, under coapplication of NIR and the DCMF.

### 3.4 Feasible Local Heating by L-MCPs under NIR Irradiation

When silica-coated particles are internalized into cells via the endocytic pathway, they typically traffic from endosomes to lysosomes.^11^ Endosomal escape is expected to be promoted by the pH-responsive surface properties, which represents a key strategy in the design of ionizable lipid-based drug delivery systems. Regarding photothermal cancer treatment therapy, including organelle dysfunction via heat stress is an effective strategy to disrupt cellular homeostasis and trigger cell death. Notably, mitochondria are often regarded as the primary organelle targets for maximizing therapeutic efficacy.^65–68^ Consequently, such localized heat stress is fatal to HeLa cells, and the aforementioned difference in cytotoxicity between L-MCPs and pristine MCPs likely stems from their distinct intracellular localization.

To interpret the differences in photothermal cytotoxicity between L-MCPs and pristine MCPs, the local temperature increase (Δ*T*) under NIR laser irradiation (808 nm) was compared across specific cellular compartments, including the ER, lysosomes, mitochondria and plasma membrane, using organelle-specific fluorescent thermometry OTGs.^39,40^ Following the internalization of particles into HeLa cells, the fluctuations in OTG fluorescence were analyzed. Upon turning on the NIR laser, the fluorescence intensity of the OTGs declined rapidly, and subsequently returned to the basal level after the laser was turned off (Figure S11 and Supporting Movie S1), successfully demonstrating particle-mediated heat generation. Notably, at around lysosomes (Figure S11b,c), the NIR-induced fluorescence decrease in the cells treated with pristine MCPs was significantly greater than that with L-MCPs. In contrast, at around mitochondria (Figure S11e,f), the fluorescence decrease mediated by L-MCPs was significantly greater than that by pristine MCPs. These distinct thermal profiles provide strong evidence of the altered intracellular particle positioning in HeLa cells.

Given that Δ*T* correlates with the number of particles localized within each organelle, both the distribution profile and the mean Δ*T* values serve as indicators of particle position within cell. Figure 6 presents the Δ*T* distributions across the ER, mitochondria, lysosomes, and the plasma membrane. The mean Δ*T* values for L-MCPs in the ER, mitochondria and plasma membrane were higher than those of pristine MCPs. Specifically, statistically significant differences in the mean Δ*T* values were observed at the mitochondria (Δ*T*_L‑MCP_ > Δ*T*_MCP_) and lysosomes (Δ*T*_L‑MCP_ < Δ*T*_MCP_). The coating layer containing cationic species facilitates attraction toward the negatively charged organelle membranes.^69^ Furthermore, branched ionizable lipids have been reported to promote endosomal escape, a key strategy widely utilized in mRNA delivery via lipid nanoparticles.^70,71^ Notably, L-MCPs enhanced the local heating at the plasma membrane. Because the extracellular tumor microenvironment is typically weakly acidic,^60^ the positive surface charge of L-MCPs is prominently enhanced under such conditions, thereby increasing their electrostatic affinity for biological membrane surface.

**Figure 6:**
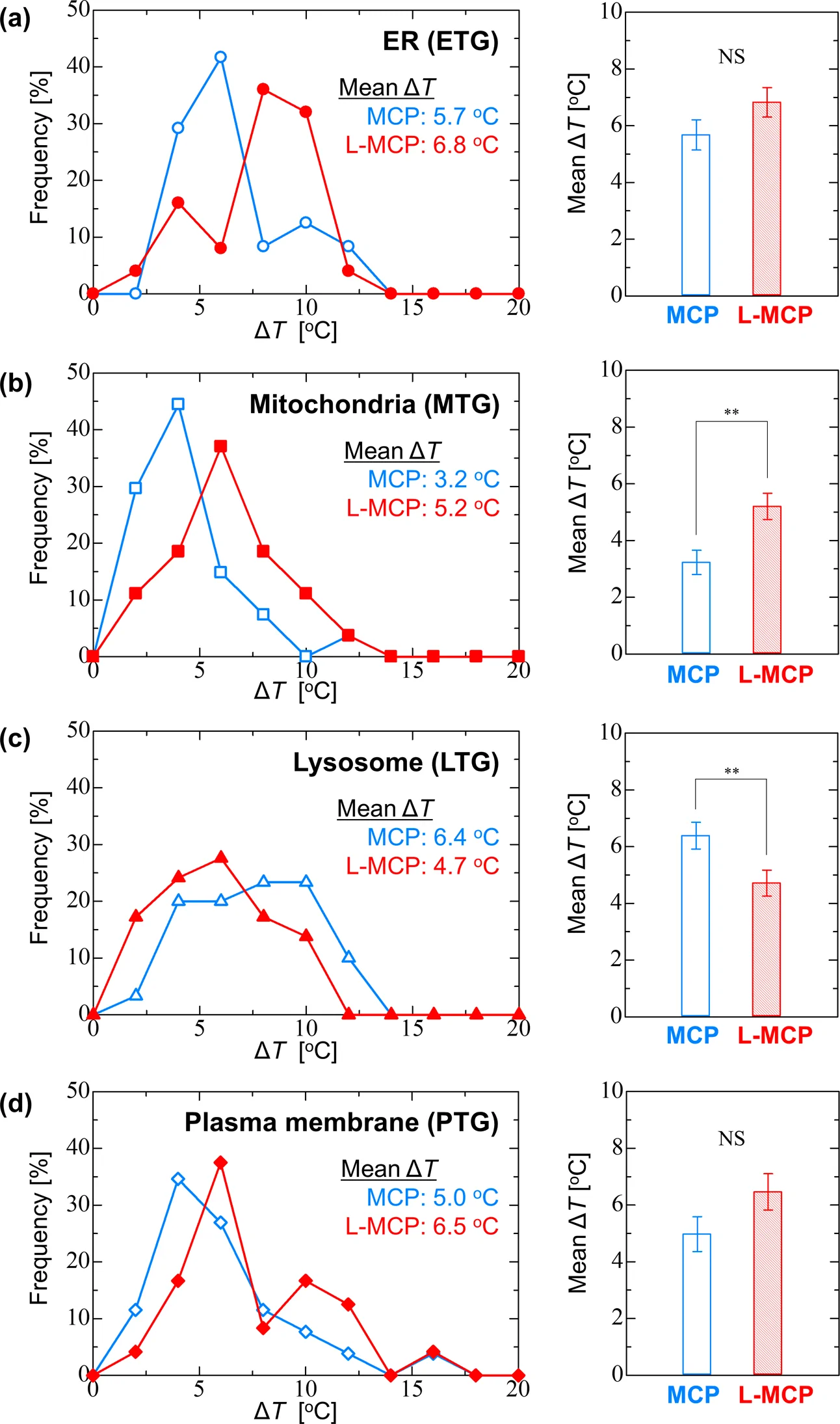
Intracellular Δ*T* distribution observed at each organelle and comparison of the mean Δ*T* values. (a) ER (ETG), (b) mitochondria (MTG), (c) lysosomes (LTG), and (d) plasma membrane (PTG). The mean Δ*T* value for each compartment was determined from a minimum of 20 independent localized spots. Statistical significance was determined by Student’s *t*-test (**p < 0.01 and NS: *p* > 0.05).

## 4 Conclusions

Functionalization of photothermal magnetic composite particles (MCPs) was successfully achieved by using the ionizable cationic lipid SM-102. Via liposome studies, the fundamental properties of SM-102-incorporated membranes were investigated. These findings were subsequently utilized to design the membrane properties of the lipid-coated particles; specifically, an SM-102/DOPC (3:7 molar ratio) provided high membrane fluidity, excellent particle dispersibility in PBS, and pH responsiveness. This ionizable lipid-based membrane coating facilitated enhanced cellular uptake in HeLa cells at pH 6.7 (a weakly acidic condition). Under combined NIR irradiation and DCMF application, the lipid-coated MCPs exhibited greater photothermal cytotoxicity than the pristine MCPs despite their comparable uptake levels. This discrepancy indicates that the lipid coating alters the intracellular localization of the particles after endocytosis. Under NIR irradiation, the local heating behaviors of the internalized particles were investigated. The pristine MCPs, which possess a silica-like surface, led to a predominant temperature increase at around lysosomes. In contrast, for lipid coated MCPs, pronounced temperature rises were observed at the ER, mitochondria, and the plasma membrane. Consequently, these findings demonstrate that lipid membranes containing the ionizable cationic lipid SM-102 possess the potential to alter the intracellular trafficking and localization of coated inorganic materials.

Designing bioactive interfaces remains a significant challenge. Although cationic lipids are powerful tools for drug delivery, their potent cytotoxicity remains a major concerned^7^ (as discussed in Section 3.3), which is primarily attributed to the strong interaction of quaternary ammonium groups with the cell membrane.^44^ Conversely, ionizable cationic lipids can mitigate cytotoxicity while offering advantages in pH-responsive cellular uptake (Section 3.3), enhanced endosomal escape,^36,70,71^ and organelle-specific delivery (Section 3.4). Furthermore, the targeted intracellular delivery of superparamagnetic iron oxide nanoparticles will facilitate a strategic transition from conventional cancer chemotherapy to advanced therapies, such as photothermal therapy^8^ and ferroptosis-based therapy.^11^ Drawing inspiration from ionizable lipid-based nanocarrier designs, this strategy offers a versatile platform for further functionalization and intracellular targeting of inorganic nanoparticles.

## Supplementary Material





## Notes

Time course of the fluorescence intensity of OTGs upon stepwise NIR irradiation (Movie S1) (MP4)
Comparison of the properties of the MCPs (Table S1); lipid bilayer thickness evaluated by DPH fluorescence quenching method (Table S2); synthetic procedure of lipid coated MCPs and effect of the *A*_liposome_/*A*_particle_ ratio on surface coverage (Figure S1); ζ potentials of SM-102/DOPC liposomes and maximum adsorption amount of TNS onto SM-102/DOPC liposomes (Figure S2); characterization of the synthesized particles (Figure S3); pH-dependent ζ potentials of the free suspended liposomes (Figure S4); photothermal performance of pristine MCPs (Figure S5); optical microscopy observation of MCPs (Figure S6); mass fraction of MNPs incorporated into MCPs and photothermal conversion efficiency per unit mass (Figure S7); schematic illustration of the SM-102/DOPC (3:7 molar ratio) bilayer (Figure S8); distributions of luminance (particle) observed inside HeLa cells (Figure S9); cell viability in the presence of DOTAP-coated MCPs (Figure S10); and fluorescence decrease/recovery of LTG by turning on/off NIR (Figure S11) (PDF)
