# Supplementary material for: Characterization of Ionizable Lipid Membranes for Designing Bioactive Interface of Inorganic Particles
Source: Langmuir. 2026 Sep 23;42(39):29532–43. doi: 10.1021/acs.langmuir.6c04968 (PMC13648268; doi:10.1021/acs.langmuir.6c04968)
Supplement: Supplementary file 1 [file la6c04968_si_002.pdf]

Supporting Information

Characterization of Ionizable Lipid Membranes for Designing Bioactive Interface of Inorganic Particles

Taisei Suzuki,[a] Keishi Suga,[a] Ikumi Kato,[a] Takeru Yamazaki,[b] Kanako Watanabe,[a] Tom A.J. Welling,[a,c] Hikaru Namigata,[a] Noriko Yamauchi,[d] Kumiko Hayashi,[e] Young-Tae Chang,[f] Satoshi Arai,[b] Daisuke Nagao[a]**

[a] Department of Chemical Engineering, Tohoku University, 6-6-07, Aoba, Aramaki-aza, Aoba-ku, Sendai 9808579, Japan

[b] Nano Life Science Institute (WPI-NanoLSI), Kanazawa University, Kakuma-machi, Kanazawa 9201192, Japan

[c] Frontier Research Institute for Interdisciplinary Sciences, Tohoku University, 6-3, Aoba, Aramaki-aza, Aoba-ku, Sendai 9808579, Japan

[d] Domain of Materials Science and Engineering, Faculty of Applied Science and Engineering, Ibaraki University, 4-12-1, Nakanarusawa-cho, Hitachi 316-8511, Japan

[e] The Institute for Solid State Physics, The University of Tokyo, 5-1-5 Kashiwano-Ha, Kashiwa 277-8581, Japan

[f] Department of Chemistry, Pohang University of Science and Technology, Pohang 37673, Republic of Korea

Corresponding authors

*K.S. (keishi.suga.b7@tohoku.ac.jp), D.N. (dnagao@tohoku.ac.jp)

Materials

Iron (III) chloride ($FeCl_3$, purity ≥95%), tetraethyl orthosilicate (TEOS, purity ≥95%), ethanol (purity ≥99.5%), chloroform (purity ≥99%), citric acid (purity ≥98%), disodium hydrogenphosphate 12-water (purity ≥98%), and 25% ammonia solution were purchased from Fujifilm Wako Chemical Corporation (Osaka, Japan). Rhodamine B isothiocyanate (RBITC) was purchased from Sigma-Aldrich (Tokyo, Japan). Iron (II) ($FeCl_2$, purity ≥99.9%) was purchased from Kojundo Chemical Laboratory Co., Ltd. (Saitama, Japan). *N*-Trimethoxysilylpropyl-*N*,*N*,*N*-trimethylammonium chloride (TSA, 50% in methanol) was purchased from Gelest Inc. (Morrisville, PA, USA). 3-Methacryloxypropyltrimethoxysilane (MPTMS, purity ≥95%) was obtained from Shin-Etsu Chemical Co., Ltd. (Tokyo, Japan). Heptadecan-9-yl-8-((2-hydroxyethyl)(6-oxo-6-(undecyloxy)hexyl)amino)octanoate (SM-102, purity ≥98%) was obtained from MedChemExpress (Monmouth Junction, NJ). 1,2-Dioleoyl-*sn*-glycero-3-phosphocholine (DOPC, purity ≥99%) and 1,2-dipalmitoyl-*sn*-glycero-3-phosphocholine (DPPC, purity ≥99%) were obtained from Yuka Sangyo Co., Ltd. (Tokyo, Japan). 1,2-Dioleoyl-3-trimethylammonium propane (DOTAP, purity ≥99%) was obtained from Merck KGaA (Darmstadt, Germany). Chemical structures of the OTGs are shown below: (a) ETG (ER), (b) LTG (lysosomes), (c) MTG (mitochondria), and (d) PTG (plasma membrane).

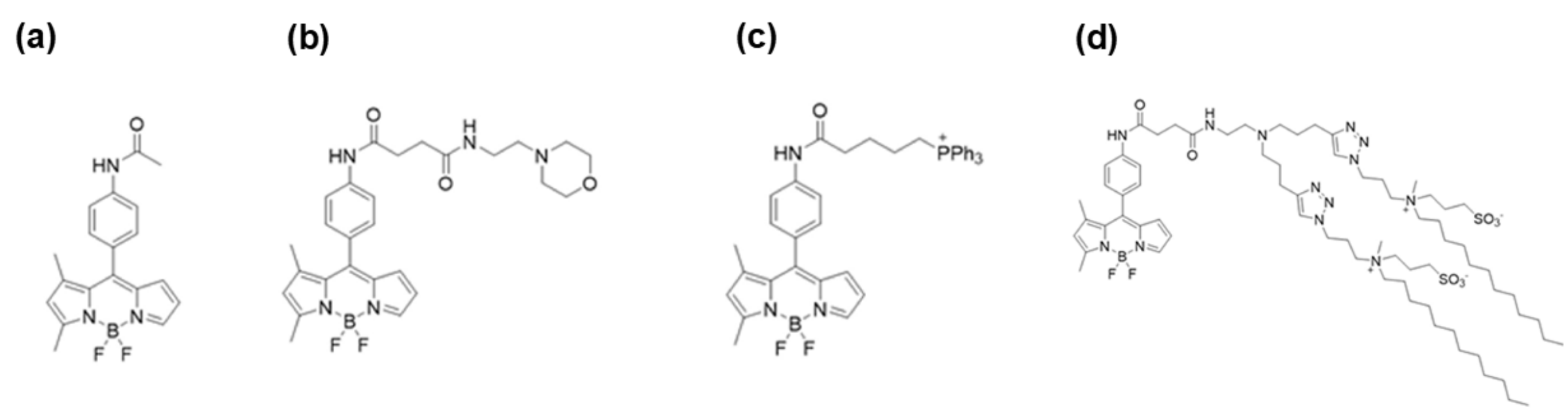

Phosphate buffer saline (PBS) was obtained from Thermo Fisher Scientific (Waltham, MA). Dulbecco's modified Eagle's medium (DMEM), fetal bovine serum (FBS), penicillin-streptomycin, and Hank's Balanced Salt Solution were purchased from Thermo Fisher Scientific (Waltham, MA, USA). Water was deionized in advance (> 18.2 MΩ cm).

Synthesis of core silica particles

Silica (SiO_2) particles of different sizes were synthesized via a sol-gel method.[1–3] The total reaction volume was 50 cm^3, and the reaction medium was composed of water/ethanol mixture. The concentrations of reagents were [TEOS] =200 mol/m^3, [NH_3] =300 mol/m^3, [H_2O] =5.5 $kmol/m^3$. After reaction, the particle suspension was washed by centrifugation (10000 × g for 15 min with ethanol, twice) and then redispersed in deionized water. The synthesized SiO_2 were characterized using a transmission electron microscope (TEM, Hitachi, HD-2700).

Synthesis of magnetic nanoparticles (MNPs)

Magnetic nanoparticles (MNPs) were prepared using a modified Massart method.[4,5] Briefly, MNPs were synthesized via the coprecipitation method using $FeCl_3$ and $FeCl_2$. Thirty seconds after initiating the coprecipitation reaction, TSA was added to the nanoparticle suspension. The total reaction volume was 250 cm^3, and the concentrations of reagents were [$FeCl_2$] =40 mol/m^3, [$FeCl_3$] =80 mol/m^3, [NH_3] = 0.70 mol/m^3, [TSA] =15 mol/m^3. After reaction, the particle suspension was washed with ethanol (centrifugation at 22000 × g for 15 min, twice) and subsequently redispersed in deionized water. The synthesized MNPs were characterized using TEM and a vibrating sample magnetometer (VSM, Toei Scientific Industrial Co., PV-M20-5). The

VSM samples were vacuum dried for at least 12 h, and the measurement range was -10000 to +10000 Oe. The saturation magnetization value was calculated using the arithmetic mean of the absolute values of the maximum and minimum magnetization values within the range.

Fabrication of MCPs

MCPs were prepared in two steps: (i) hetero-coagulation and (ii) silica coating, based on a previously reported method. First, MNPs were deposited on the SiO_2 particles by mixing the suspensions at 25°C for 1 min. The total reaction volume was 20 cm^3, and the concentrations (volume fraction in mixture) of MNPs and SiO_2 were 0.092 and 0.034 vol%, respectively. Excess MNPs were removed via centrifugation (14000 × g for 10 min, three times). The coagulated particles (SiO_2@MNP) were redispersed in water. Next, coagulated particles were coated with silica. The particle suspensions were sonicated for 1.5 h. TEOS, MPTMS, RBITC, ammonia solution, ethanol and deionized water were then added to initiate the silica-coating reaction. The total reaction volume was 20 cm^3, and the concentrations of reagents were [TEOS] = 25 mol/m^3, [MPTMS] = 25 mol/m^3, [RBITC] = 0.05 mol/m^3, [NH_3] = 300 mol/m^3, and [H_2O] = 9.0 $kmol/m^3$. The mixtures were shaken mechanically for 24 h at 25°C. The obtained MCPs were washed by centrifugation (14000 × g for 15 min with ethanol, three times) and subsequently redispersed in deionized water.

Preparation of liposomes

Liposomes were prepared by extrusion method. Briefly, lipids were dissolved in chloroform, then dried in a round-bottom flask by rotary evaporation under vacuum for at least 3 h. The

obtained lipid films were hydrated with deionized water at 60 °C. The liposome suspensions were frozen at −70 °C and thawed at 60 °C. This freeze−thaw cycle was repeated five times. The liposome suspensions were extruded 11 times through a polycarbonate membrane with a mean pore diameter of 400 nm, using an extruding device (Liposofast; Avestin Inc., Ottawa, ON, Canada). The total lipid concentration of the liposome samples was adjusted to 10 mM.

Surface coverage of particles

The total surface area of the liposomes (A_{liposome}) and core particles (A_{particle}) was calculated using the following equations (S1, S2):

$$A_{\text{liposome}} = \frac{S_{\text{lipid}}\, N_{\text{A}}\, C_{\text{lipid}}}{2} \tag{S1}$$

$$A_{\text{particle}} = \frac{6}{\rho\, d_{\text{V}}} C_{\text{particle}} \tag{S2}$$

where S_{lipid} is the mean headgroup area of a lipid molecule (S_{lipid} =0.72 nm^2 for DOPC),[6] N_{A} is Avogadro's number, C_{lipid} is the molar concentration of the lipid, ρ is the density of the core particle (ρ = 1.9 g/cm^3 for silica), d_{V} is the particle diameter determined by TEM, and C_{particle} is the mass concentration of the particles. Typically, the reaction volume was 1 mL, with C_{lipid} = 25 mM and C_{particle} = 0.02 g/mL, which corresponds to an A_{liposome} / A_{particle} ratio of 20. After extrusion, excess liposomes were removed by centrifugation (2300 × g for 5 min with deionized water) and redispersed in deionized water.

The effect of the A_{liposome} / A_{particle} ratio on surface coverage was investigated. Fluorescence-labelled DOTAP liposomes (containing 0.1 mol% of Rhodamine DHPE) were coated onto SiO_2, and the amount of adsorbed lipid was quantified (Figure S1a). Additionally, DOTAP liposomes were coated onto MCPs, and their ζ potentials were evaluated (Figure S1b). Both lipid adsorption

and ζ potential value saturated at an A_{liposome} / A_{particle} ratio of 20, suggesting sufficient lipid coating. Similarly, SM-102/DOPC (3:7 molar ratio) liposomes were coated onto particles at an A_{liposome} / A_{particle} ratio of 20.

<u>Size distribution and zeta potential measurements</u>

The hydrodynamic diameters and zeta potentials of particles were determined at 25°C using DLS. Particles were dispersed in deionized water or PBS, where the particle concentration was adjusted to 0.01 vol%.

For zeta potential measurements, the pH was adjusted using citrate-phosphate buffer (pH 3–10). pK_a value was analyzed based on the Henderson-Hasselbalch equation (S3):[7,8]

$$y = \frac{D_{\max} - D_{\min}}{1 + \exp\left(\frac{x - x'}{dx}\right)} + D_{\min} \tag{S3}$$

where $D_{\max}$, $D_{\min}$, x', dx are fitting parameters. x (pH) and y (zeta potential) are experimental parameters. pK_a value was defined as the x value fills y =0.

<u>Membrane fluidity measurements</u>

To determine membrane properties, the membrane fluidity was investigated by using a fluorescent probe DPH (1,6-diphenyl-1,3-5-hexatriene), based on previously described methods.[9–11] Fluorescence spectroscopy measurements were performed in a quartz cell (10 mm × 10 mm), using a Fluorescence spectrophotometer (F-7000 (Hitachi High Tech)), wherein the total concentrations of lipids and DPH were adjusted to 0.1 mM and 1.0 µM, respectively. The samples were excited with vertically polarized light (λ_{ex} = 360 nm) and emission intensities for both

perpendicular ($I_{\perp}$) and parallel ($I_{\parallel}$) to the excited light were recorded at 430 nm. The polarization (P_{DPH}) of DPH was then calculated by the following equations (S4, S5):

$$P_{\mathrm{DPH}} = \frac{I_{\parallel} - GI_{\perp}}{I_{\parallel} + GI_{\perp}} \tag{S4}$$

$$G = i_{\perp}/i_{\parallel} \tag{S5}$$

where $i_{\perp}$ and $i_{\parallel}$ are emission intensities perpendicular and parallel to the horizontally polarized light, respectively, and G is the correction factor. The $1/P_{\mathrm{DPH}}$, reciprocal of polarization (P_{DPH}), was calculated as the membrane fluidity. At least three measurements were carried out for each sample, and the average of $1/P_{\mathrm{DPH}}$ values and standard deviations were calculated.

Surface charge density analysis

The surface charge density of lipid membrane was based on the adsorption amount of anionic fluorescent probe TNS (6-(*p*-Toluidino)-2-naphthalenesulfonic acid sodium salt).[12,13] An aliquot of the TNS solution (100 µM in ethanol) was added to the particle suspensions containing 100 µM of lipids ([SM-102]+[DOPC]). The TNS fluorescence emission spectra (excitation wavelength 340 nm) were obtained under varying TNS concentrations using a fluorescence spectrophotometer (F-7000, Hitachi High-Tech Science Corporation, Tokyo, Japan). The peak intensities at approximately 438 nm (I_{438}) were recorded, and the adsorbed TNS concentrations (C_{ads}) were calculated as following equations (S6, S7):

$$C_{ads} = \frac{I_{438}}{I_{438,1\mu M}} \times C_{TNS} \tag{S6}$$

$$C_{TNS} = C_{ads} + C_{eq} \quad (S7)$$

where C_{TNS} and $I_{438,1\mu M}$ correspond to the TNS concentration and the fluorescence intensity at [C_{TNS}] = 1 μM, respectively, given that 1 μM of TNS was fully adsorbed by the lipid-coated particles. C_{eq} corresponds to the equilibrium concentration of TNS in the solution.

Using the obtained Langmuir plot, the binding affinity K [μM^{-1}] and saturated adsorption volume q_{max} [μmol/g] were calculated as follows (S8):

$$q = \frac{q_{max} K C_{eq}}{1 + K C_{eq}} \quad (S8)$$

At a total lipid concentration of 100 μM, Eq. S8 was modified as follows (S9):

$$\frac{1}{C_{ads}} = \frac{1}{K C_{max}} \frac{1}{C_{eq}} + \frac{1}{C_{max}} \quad (S9).$$

C_{max} values indicate the maximum number of cationic groups, which is relevant to the number density of cationic groups per unit surface area.

Membrane polarity measurements

The membrane polarity was investigated by using a fluorescent probe Laurdan (6-dodecanoyl-2-dimethylaminonaphthalene), based on previously described methods.[9,10,14] Fluorescence spectroscopy measurements were performed in a quartz cell (10 mm × 10 mm), using a Fluorescence spectrophotometer (F-7000 (Hitachi High Tech)), wherein the total concentrations of lipids and Laurdan were adjusted to 0.1 mM and 1.0 μM, respectively. The fluorescence spectrum was recorded with excitation wavelength of 340 nm, at appropriate emission wavelengths from 400 nm to 600 nm at various temperatures, and the peak intensity was normalized by

spectrum area. The membrane polarity (GP_{340}) was estimated based on the following equation (S10):

$$GP_{340} = \frac{I_{440} - I_{490}}{I_{440} + I_{490}} \qquad (S10)$$

where I_{440} and I_{490} are the emission intensities of Laurdan at 440 and 490 nm, respectively.

Magnetic properties of MCPs

The apparent mass fraction of the magnetic nanoparticles (MNPs) was estimated according to our previous method:[2] the observed saturation magnetization (M) is related to the mass fraction of the incorporated MNPs as follows (S11, S12):

$$M = M_{\mathrm{MNP}} w_{\mathrm{MNP}} + M_{\mathrm{silica}} w_{\mathrm{silica}} \qquad (S11)$$

$$w_{\mathrm{MNP}} + w_{\mathrm{silica}} = 1 \qquad (S12)$$

where M_{MNP} and M_{silica} represent the saturation magnetization of MNPs and core SiO_2 particles (M_{silica} = 0 emu/g), respectively, and w_{MNP} and w_{silica} represent the mass fractions of the MNPs and core SiO_2 particles, respectively. The specific surface area of the SiO_2 core was calculated as as $6/(\rho\, d_{\mathrm{V,silica}})$, where ρ is a density of silica (=1.9 /cm^3).

Fluorescence quenching of DPH for bilayer thickness measurements

The membrane thickness was evaluated using a fluorescence quenching method. TEMPO is a water-soluble nitroxide radical that quenches neighboring fluorescence dyes, whereas DPH is a hydrophobic dye that preferentially locates at the hydrophobic interior of the lipid bilayer.[9] Considering the partitioning behaviors of DPH and TEMPO, the closest distance between them

directly reflects the lipid bilayer thickness. The Förster radius (R_0) of DPH is reported to be 20 Å.[15] The apparent distance (r) between DPH and TEMPO can be estimated based on Förster resonance energy transfer (FRET) theory using the following equation (S13):

$$E = 1 - \frac{F}{F_0} = \frac{1}{1 + \left(\frac{r}{R_0}\right)^6} \qquad \text{(S13)}$$

where E is the energy transfer efficiency. F and F_0 represent the fluorescence intensities of DPH in the presence and absence of TEMPO. Because the distance r corresponds to the distance from the bilayer center to the outer leaflet, the total bilayer thickness is estimated as $2r$.

The steady-state fluorescence quenching of DPH by TEMPO was evaluated. A 1 mL of DPH-labeled liposome suspension ([total lipid] = 100 μM, [DPH] = 0.025 μM) was incubated at room temperature for 30 min to ensure complete insertion and equilibration of DPH within the hydrophobic interior of the lipid bilayer. Subsequently, 20 μL of TEMPO solution ([TEMPO] = 500 mM, in ethanol) was added to each suspension. Following incubation at room temperature for 10 min, the steady-state fluorescence spectrum of DPH was recorded at excitation and emission wavelengths of 360 nm and 430 nm, respectively. All measurements were conducted triplicate (n = 3) and values are expressed as mean ± standard error (SE).

The measured bilayer thicknesses ($2r$) of DOPC (l_d phase at 21°C) and DPPC (s_o phase at 21°C) liposomes were 32.0 ± 0.4 Å and 46.1 ± 4.2 Å, respectively (Table S2). These experimental $2r$ values agree well with literature values for the hydrocarbon core thickness (29–34 Å for DOPC[16,17] and 39–45 Å for DPPC in s_o phase[17,18]), validating the accuracy of this fluorescence quenching methodology. Furthermore, the $2r$ values of SM-102/DOPC (3:7 molar ratio) liposomes and L-SiO_2 were determined 31.0 ± 0.5 Å and 33.2±0.6 Å, respectively (Table S2).

Table S1 Comparison of the properties of the MCPs (Kameda et al,[2] in this study)

	Core SiO_2 diameter $d_{V,silica}$ [nm]	MCP diameter $d_{V,MCP}$ [nm]	Saturation magnetization M [emu/g]	w_{MNP} [-][*d]	Shell thickness [nm][*e]
MCP1 [*a]	182	206	23.4	0.358	12.0
MCP2 [*a]	274	312	19.5	0.298	20.5
MCP3 [*a]	422	451	10.5	0.161	14.5
MCP [*b]	199	229	18.0	0.260	15.0
MCP [*c]	372	402	12.0	0.206	15.0

[*a] data from Kameda et al.,[2] using MNPs with a saturation magnetization of 65.4 emu/g.

[*b] data from this study, using MNPs with a saturation magnetization of 69.1 emu/g

[*c] data from this study (not cited in the main text), using MNPs with a saturation magnetization of 65.4 emu/g

[*d] calculated by eqs. S11 and S12

[*e] calculated by $(d_{V,MCP} - d_{V,silica})$ /2

Table S2 Lipid bilayer thickness evaluated by DPH fluorescence quenching method

	F/F_0 [-]	E [-]	r [Å]	Bilayer thickness $2r$ [Å]
DOPC	0.209 ± 0.011	0.791	16.0	32.0 ± 0.4
DPPC	0.687 ± 0.099	0.313	23.2	46.4 ± 4.1
SM-102/DOPC (3:7)	0.178 ± 0.014	0.822	15.5	31.0 ± 0.5
L-SiO_2	0.246 ± 0.021	0.754	16.6	33.2 ± 0.6

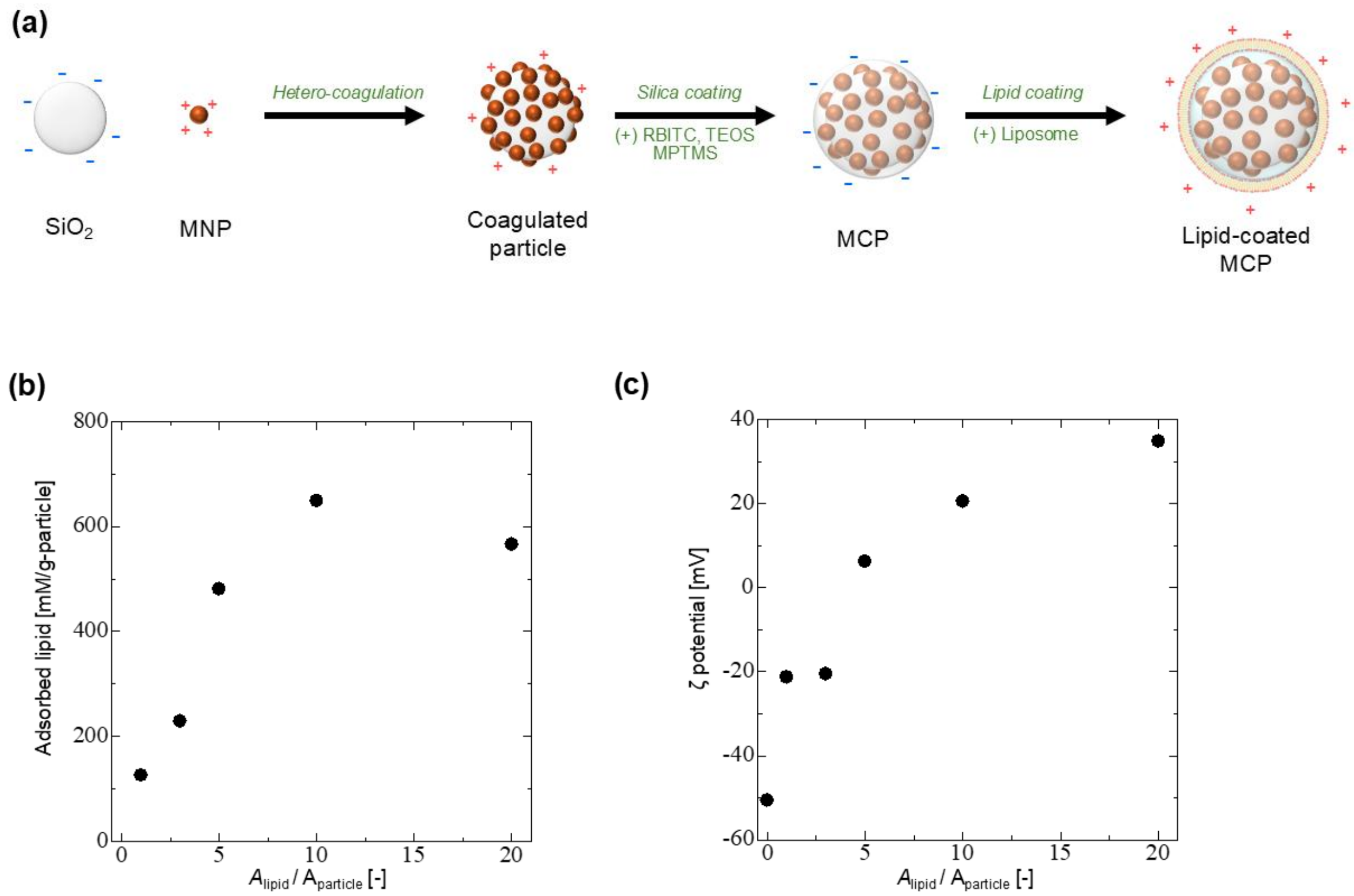

Figure S1. (a) Synthetic procedure of lipid coated MCPs. (b, c) Effect of A_{liposome} / A_{particle} ratio on surface coverage: (b) adsorbed amount of DOTAP lipid on SiO_2; (c) ζ potentials of DOTAP-coated MCPs.

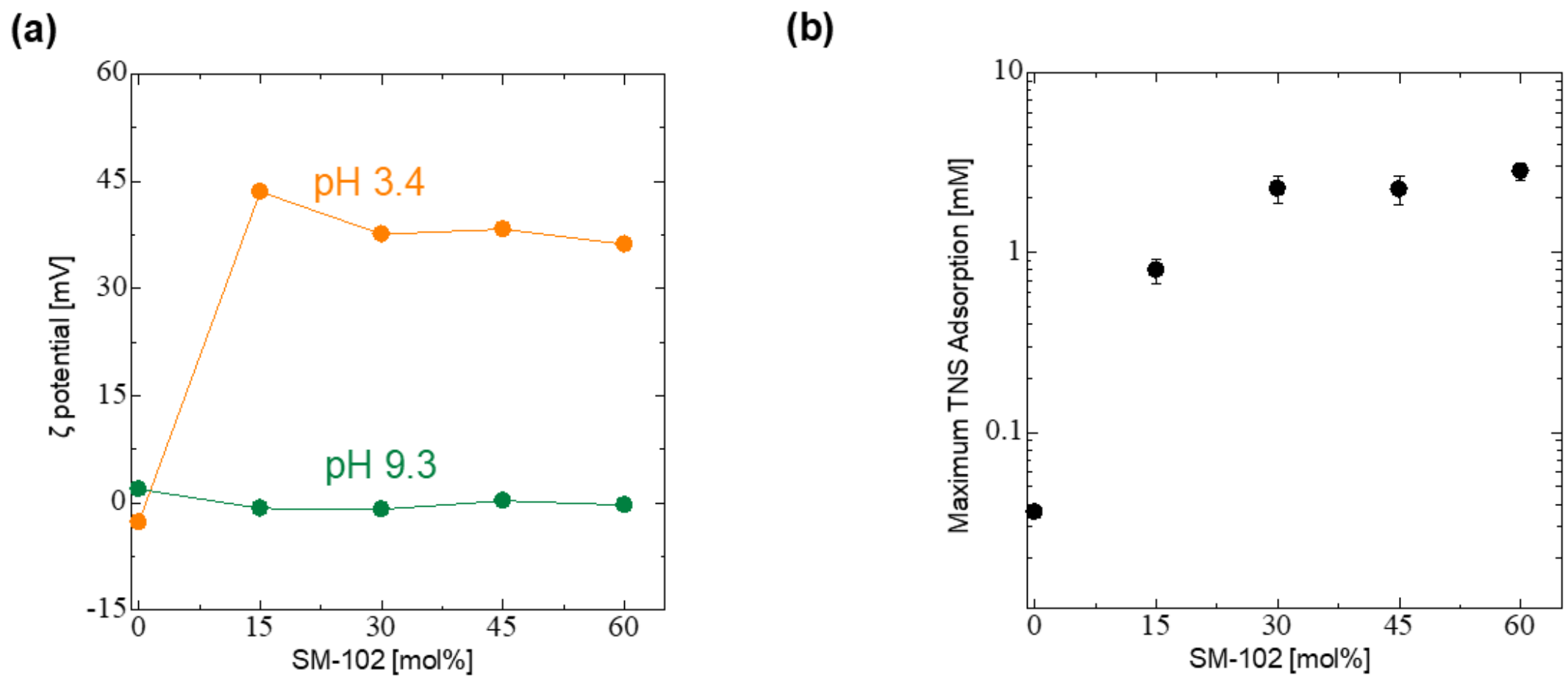

Figure S2. (a) ζ potentials of SM-102/DOPC liposomes at various molar ratio, measured at pH 3.4 or 9.3. (b) Maximum adsorption amount of TNS onto SM-102/DOPC liposomes.

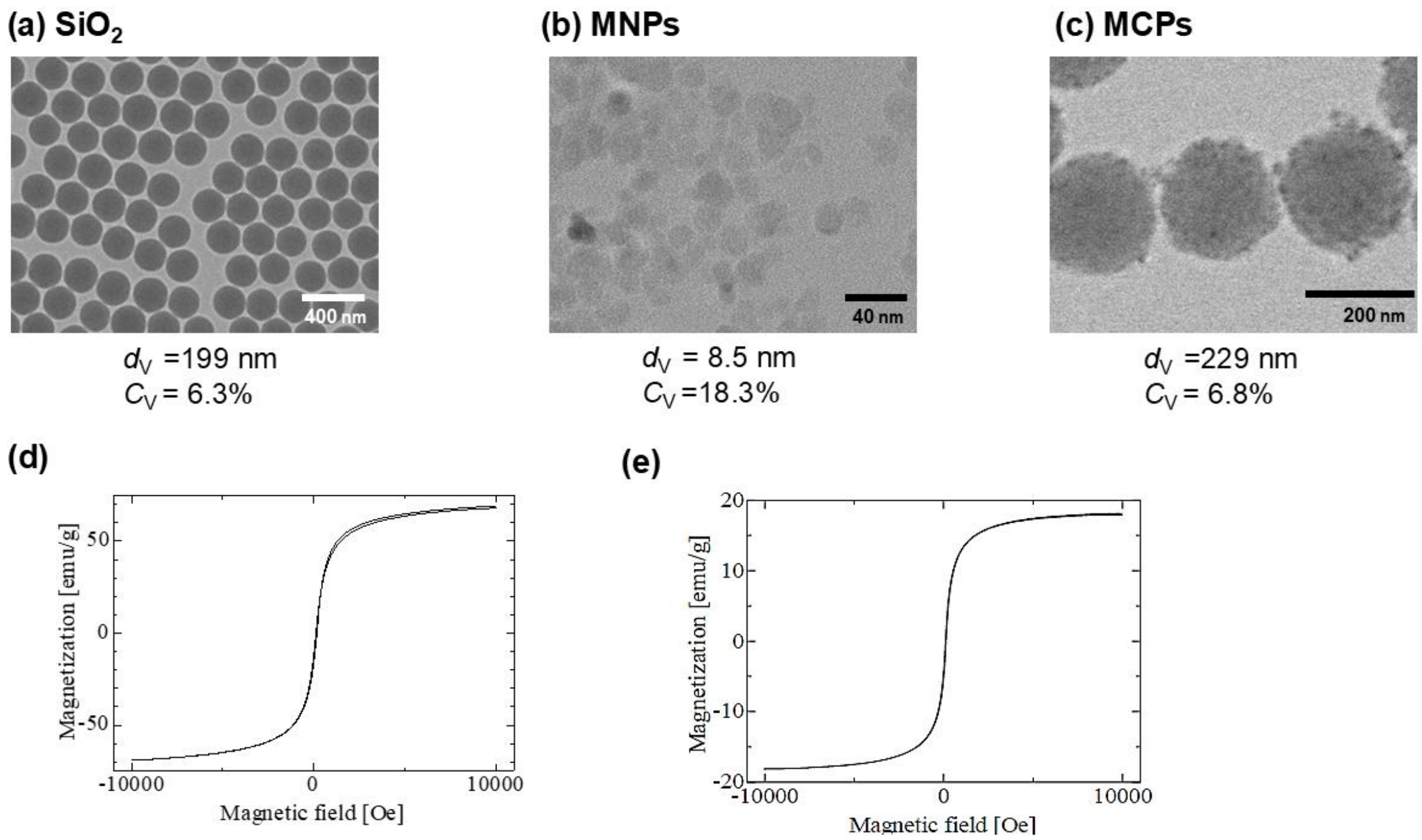

Figure S3 TEM images of the particles: (a) SiO_2 (d_v =199 nm); (b) MNPs (d_v =8.5 nm); (c) MCPs (d_v =229 nm)). Magnetization curves of particles: (d) MNPs; (e) pristine MCPs. The saturation magnetizations of MNPs and MCPs are 69.1 and 18.0 emu/g, respectively.

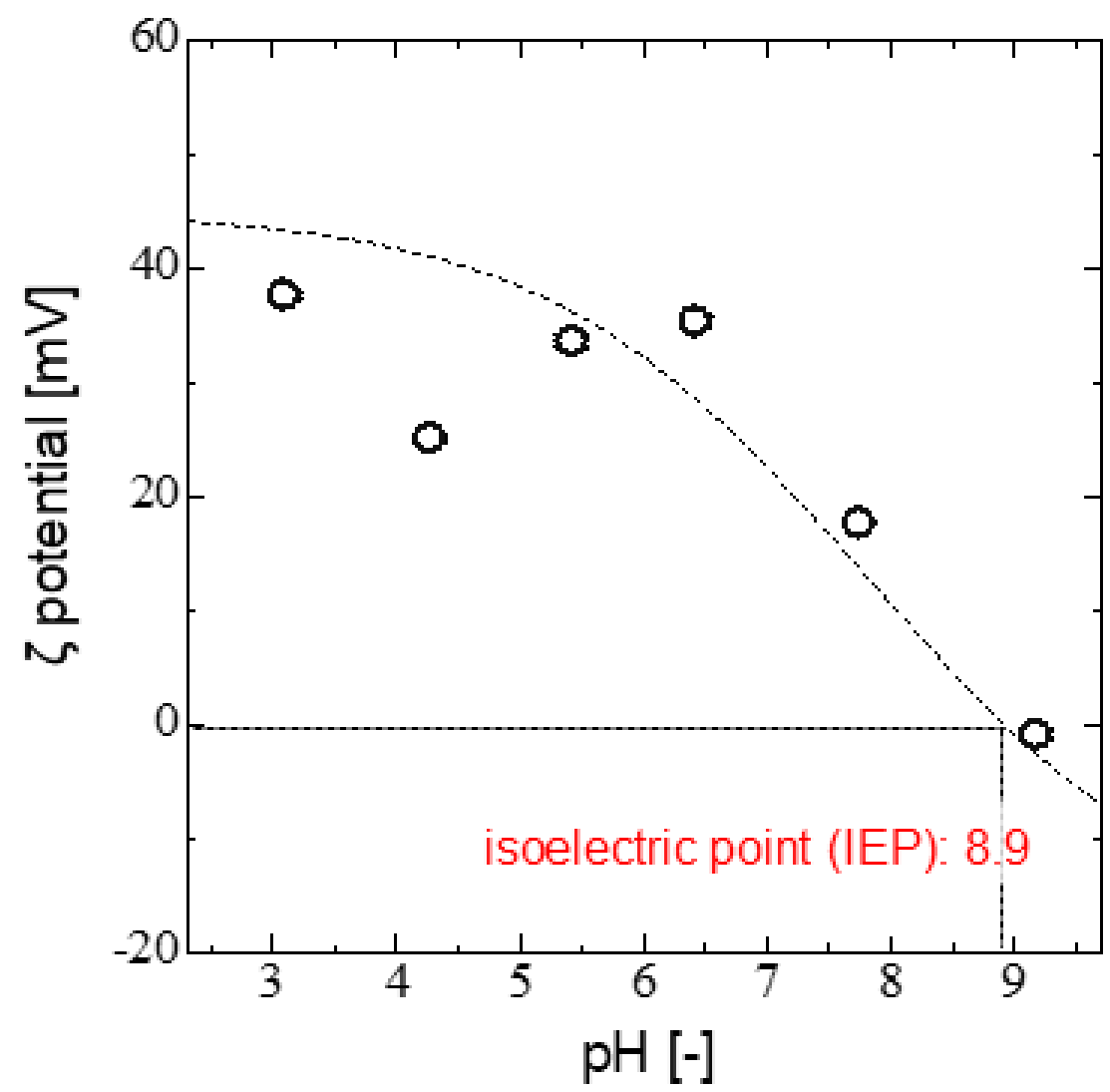

Figure S4. pH-dependent ζ potentials of the free suspending liposomes (SM-102/DOPC (3:7 molar ratio).

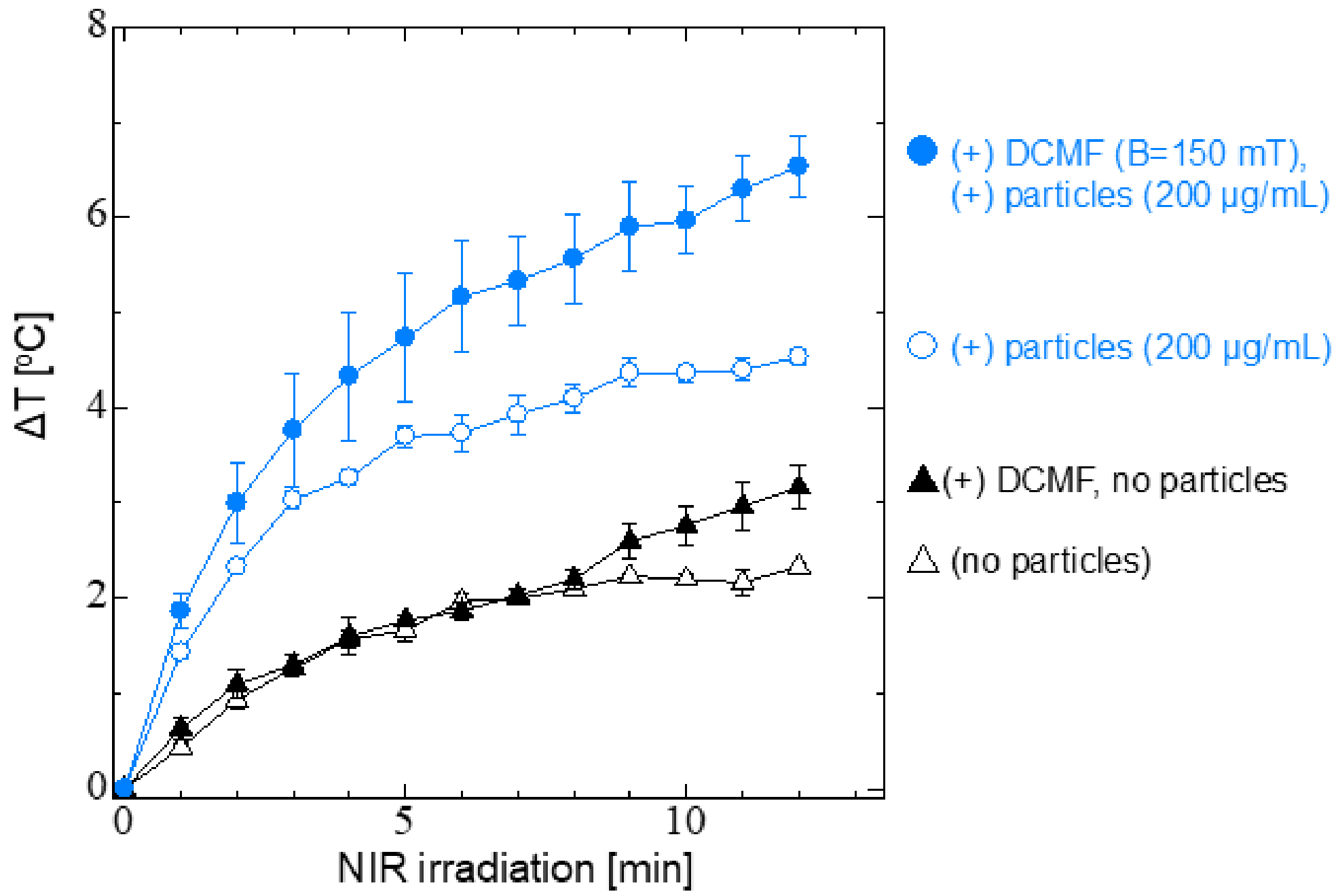

Figure S5 Photothermal performance of pristine MCPs under NIR irradiation (λ = 808 nm, 5 W/cm^2), without or with a DCMF (*B* = 150 mT). Symbols indicate the control (PBS only, open triangles), only with the DCMF (filled triangles), MCP with NIR (open circle), and MCP with NIR and with DCMF (*B* =150 mT) (filled circle).

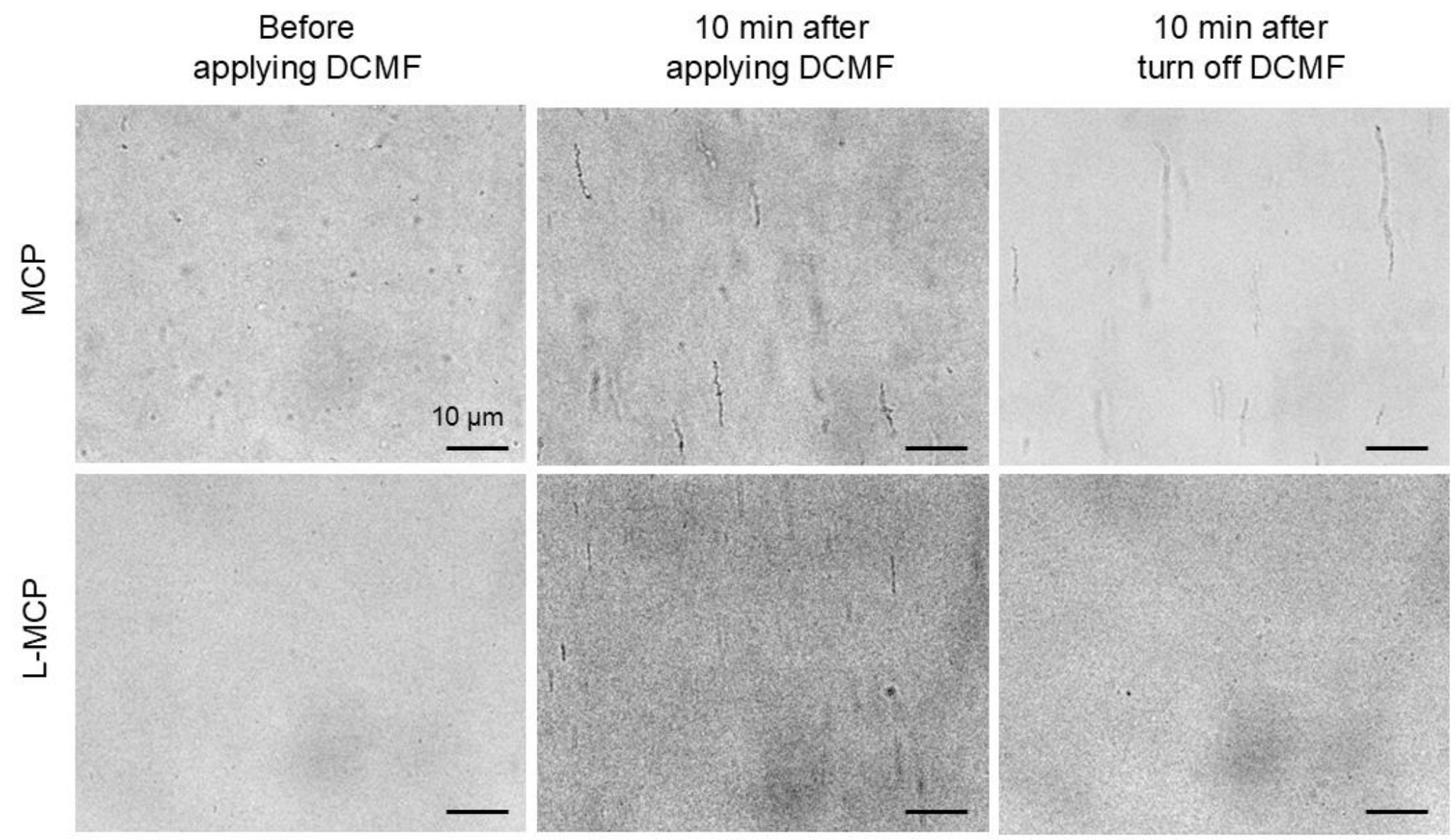

Figure S6. Optical microscopic images of MCPs and L-MCPs. Chain-like clusters were formed by applying a direct current magnetic field (DCMF, B = 150 mT) for 10 min.

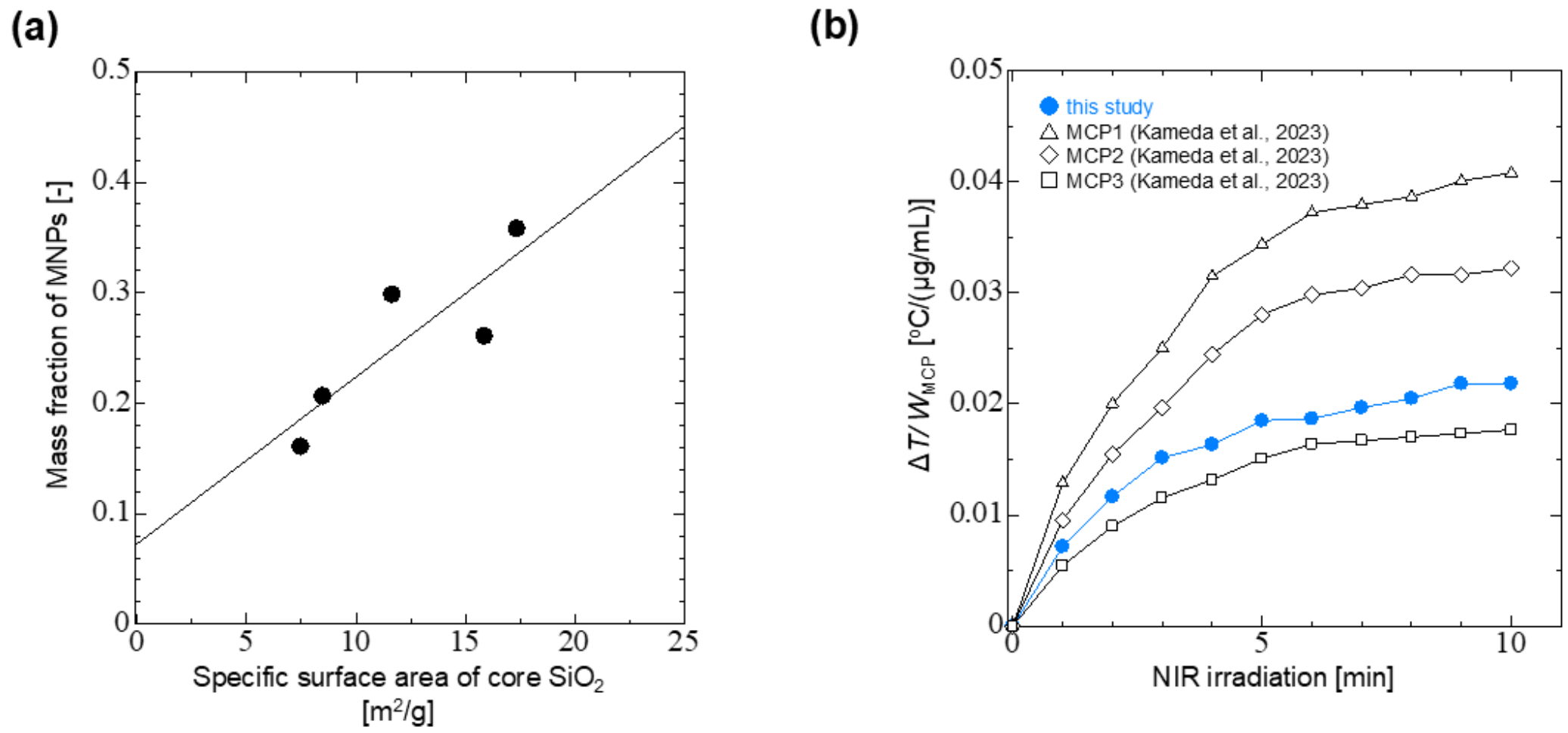

Figure S7. (a) Mass fraction of MNPs incorporated into MCPs fabricated using SiO_2 cores of different sizes. x-axis is a specific surface area calculated by $6/(\rho d_{\mathrm{V,silica}})$. (b) Photothermal conversion efficiency per unit mass. The increase in temperature (ΔT) is normalized by the applied MCP concentration (W_{MCP} [μg/mL]. Data for MCP1, MCP2, and MCP3 are adapted from our previous report (Kameda et al., 2023).[2]

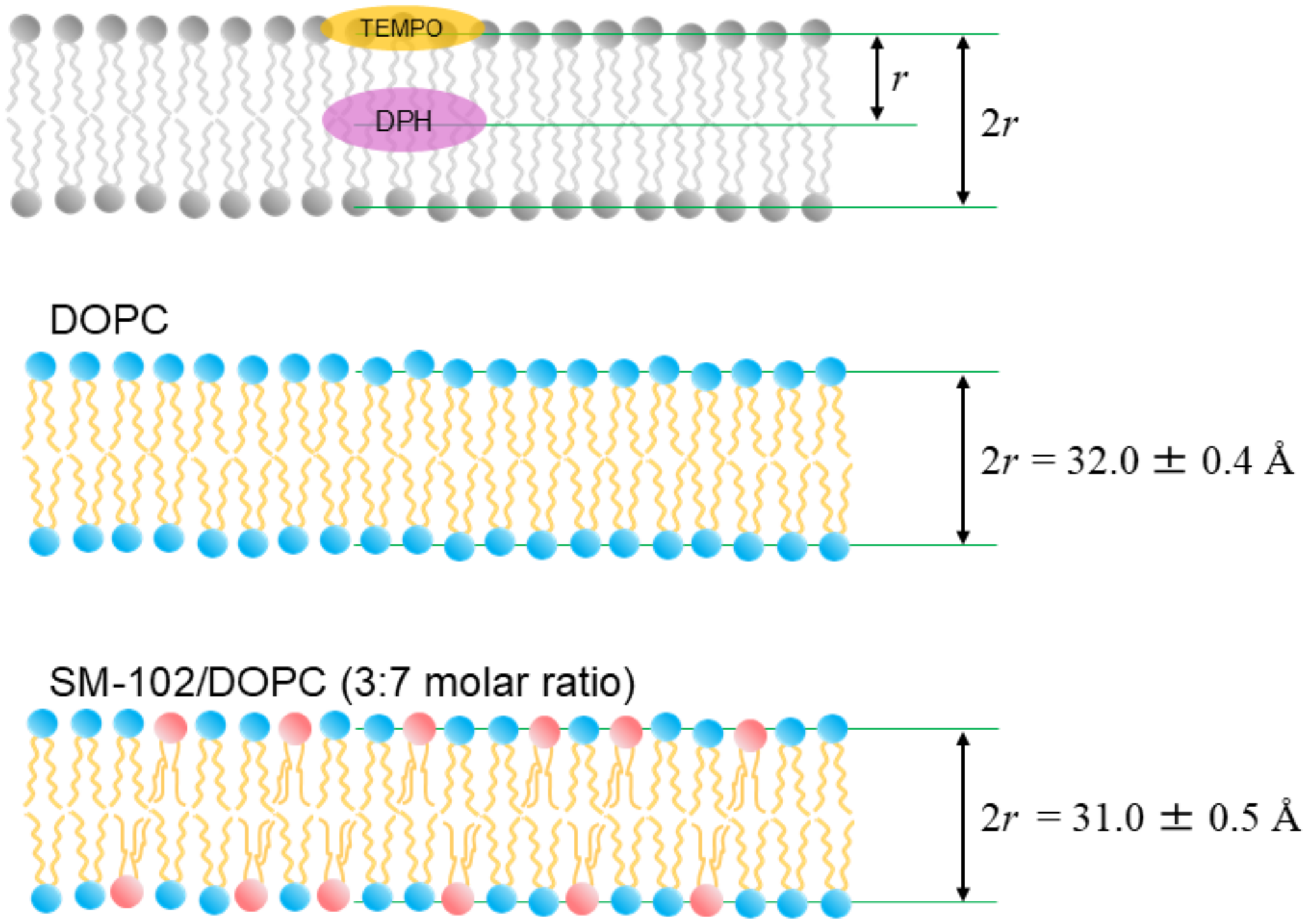

Figure S8. Schematic illustration of SM-102/DOPC (3:7 molar ratio) bilayer. Hydrocarbon core thickness (2*r*) was evaluated by DPH fluorescence quenching method.

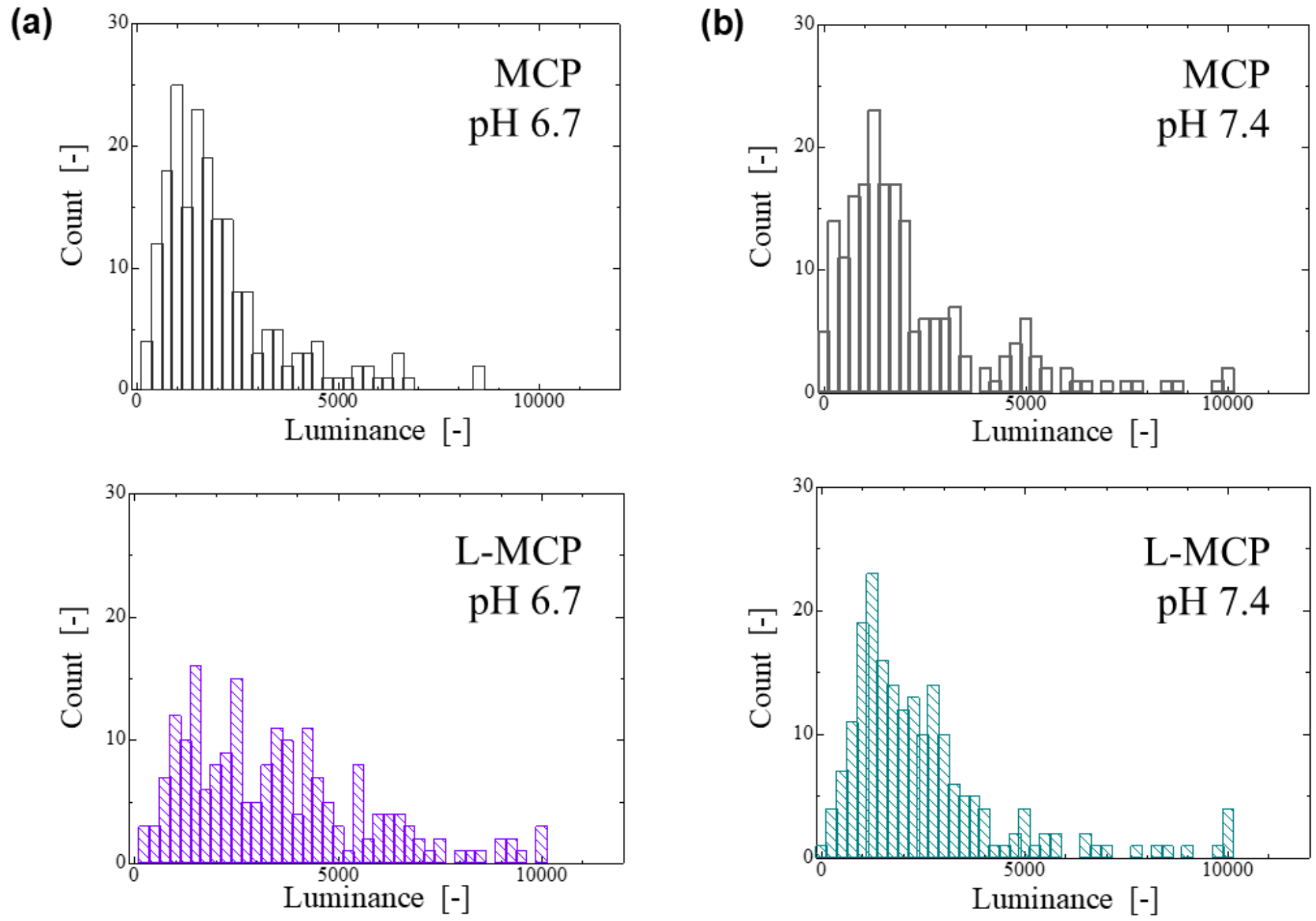

Figure S9 Distributions of luminance (which correlates with the number of particles) observed inside HeLa cells. (a) pH 6.7, (b) pH 7.4. The applied particle concentration was 200 μg/mL. At least 200 cells were analyzed to obtain each histogram.

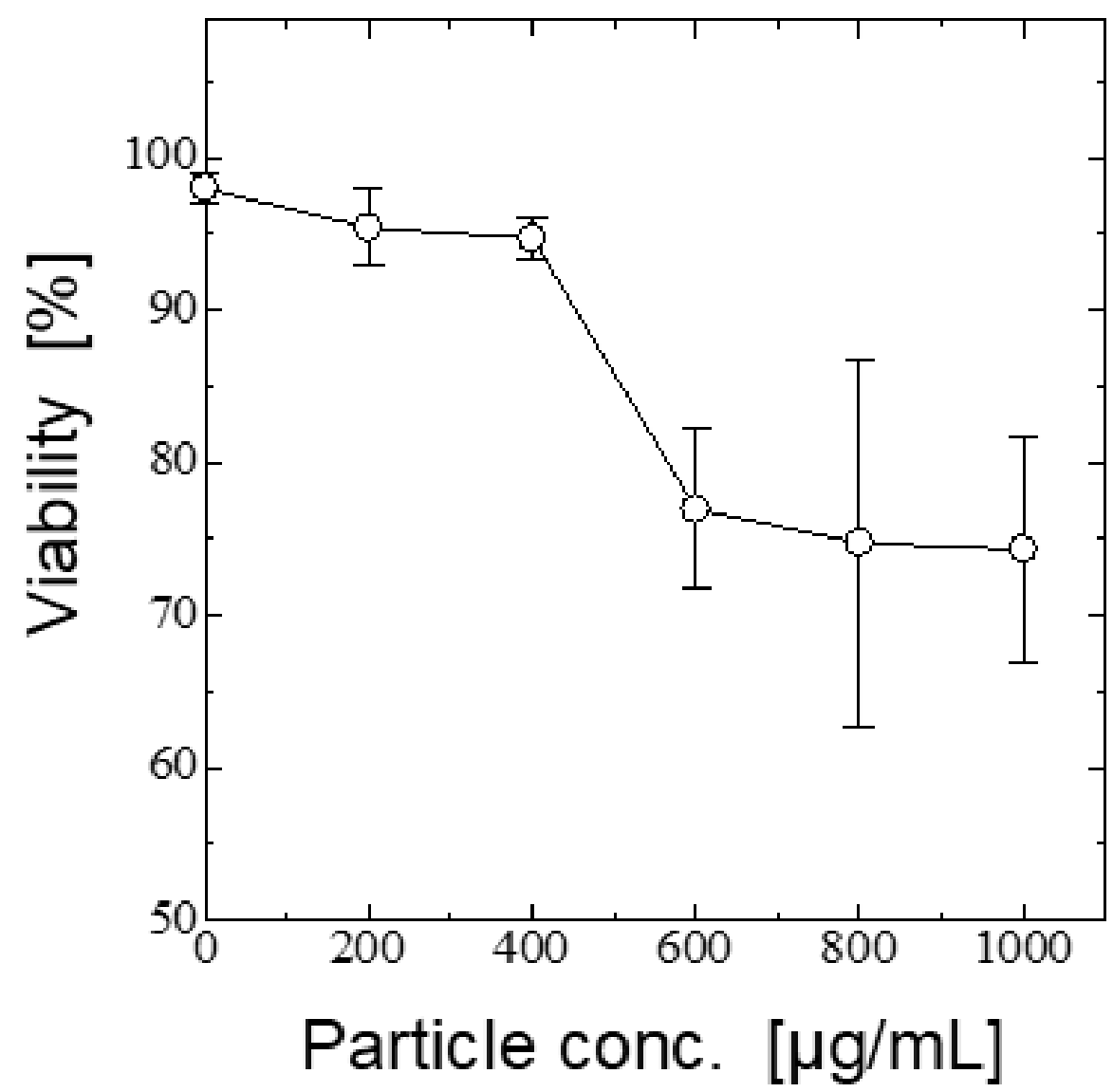

Figure S10 Cell viability in the presence of DOTAP-coated MCPs.

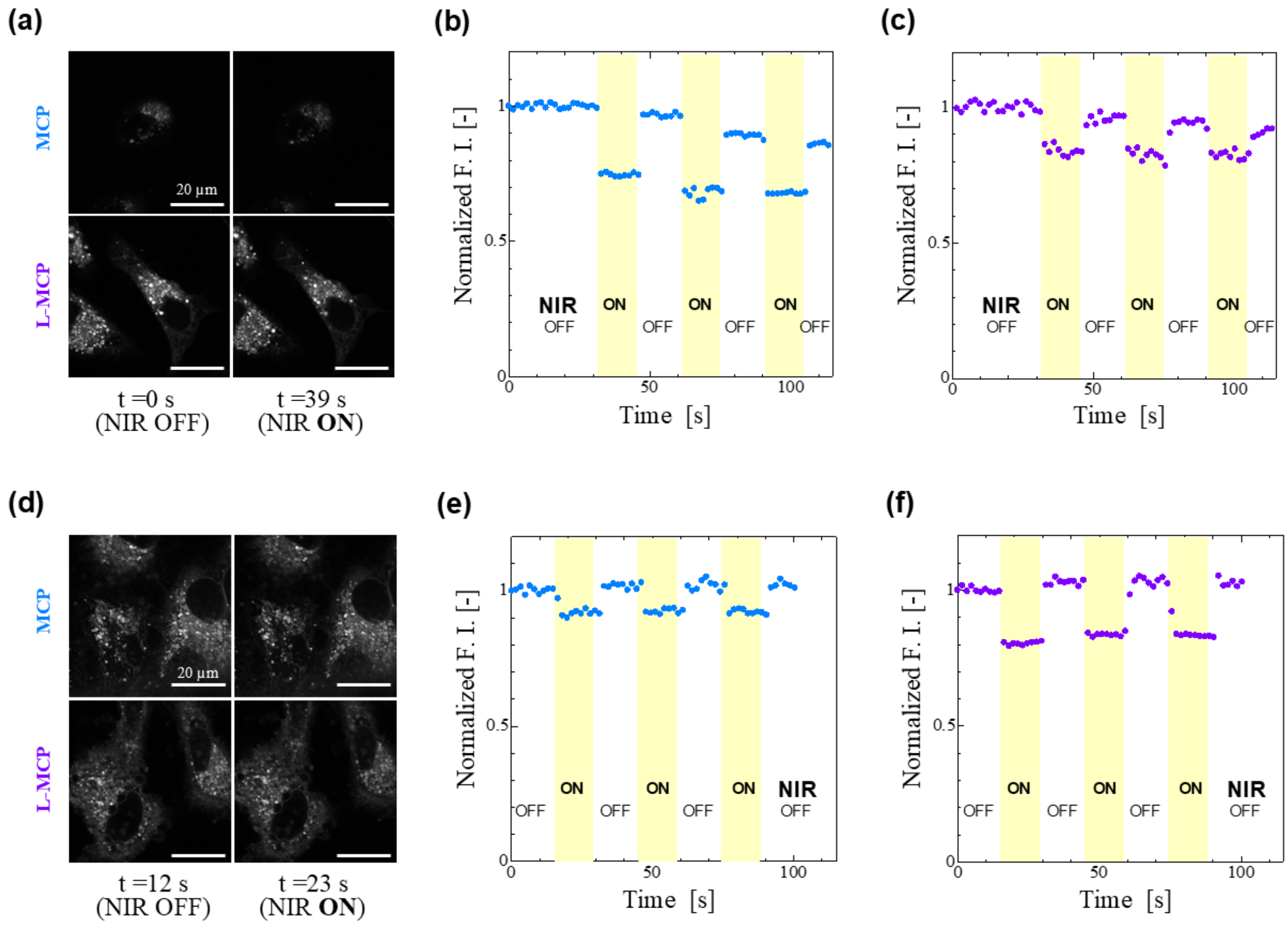

Figure S11 CLSM images and fluorescence response of OTGs to NIR irradiation, in the presence of MCPs or L-MCPs. (a) CLSM images of LTG (lysosome) in HeLa cells. (b) Fluorescence response of LTG in the presence of MCPs. (c) Fluorescence response of LTG in the presence of L-MCPs. (d) CLSM images of MTG (mitochondria) in HeLa cells. (e) Fluorescence response of MTG in the presence of MCPs. (f) Fluorescence response of MTG in the presence of L-MCPs. Scale bar: 20 µm.

References

(1) Stöber, W.; Fink, A.; Bohn, E. Controlled Growth of Monodisperse Silica Spheres in the Micron Size Range. *J. Colloid Interface Sci.* **1968**, *26* (1), 62–69. https://doi.org/10.1016/0021-9797(68)90272-5.

(2) Kameda, R.; Suzuki, T.; Kato, I.; Suga, K.; Watanabe, K.; Nagino, K.; Hayashi, K.; Arai, S.; Nagao, D. Intracellular Magnetic Control over the Reversible Assembly of Silica-Based Magnetic Composite Particles for Photothermal Therapy. *ACS Appl. Nano Mater.* **2023**, *6* (5), 3873–3883. https://doi.org/10.1021/ACSANM.2C05550.

(3) Mochizuki, T.; Sampei, S.; Suga, K.; Watanabe, K.; Welling, T. A. J.; Nagao, D. A Quantitative Approach to Characterize the Surface Modification on Nanoparticles Based on Localized Dielectric Environments. *Anal. Chem.* **2024**, *96* (8), 3284–3290. https://doi.org/10.1021/acs.analchem.3c03593.

(4) Massart, R. Preparation of Aqueous Magnetic Liquids in Alkaline and Acidic Media. *IEEE Trans. Magn.* **1981**, *17* (2), 1247–1248. https://doi.org/10.1109/TMAG.1981.1061188.

(5) Matsumoto, H.; Nagao, D.; Konno, M. Repetitive Heterocoagulation of Oppositely Charged Particles for Enhancement of Magnetic Nanoparticle Loading into Monodisperse Silica Particles. *Langmuir* **2010**, *26* (6), 4207–4211. https://doi.org/10.1021/la903266e.

(6) Liu, Y.; Nagle, J. F. Diffuse Scattering Provides Material Parameters and Electron Density Profiles of Biomembranes. *Phys. Rev. E* **2004**, *69* (4), 040901. https://doi.org/10.1103/PhysRevE.69.040901.

(7) Zuidam, N. J.; Barenholz, Y. Electrostatic Parameters of Cationic Liposomes Commonly Used for Gene Delivery as Determined by 4-Heptadecyl-7-Hydroxycoumarin. *Biochim. Biophys. Acta - Biomembr.* **1997**, *1329* (2), 211–222. https://doi.org/10.1016/S0005-2736(97)00110-7.

(8) Suga, K.; Akizaki, K.; Umakoshi, H. Quantitative Monitoring of Microphase Separation Behaviors in Cationic Liposomes Using HHC, DPH, and Laurdan: Estimation of the Local Electrostatic Potentials in Microdomains. *Langmuir* **2016**, *32* (15). https://doi.org/10.1021/acs.langmuir.5b04682.

(9) Suga, K.; Umakoshi, H. Detection of Nanosized Ordered Domains in DOPC/DPPC and DOPC/CH Binary Lipid Mixture Systems of Large Unilamellar Vesicles Using a TEMPO Quenching Method. *Langmuir* **2013**, *29* (15), 4830–4838. https://doi.org/10.1021/la304768f.

(10) Suga, K.; Matsui, D.; Watanabe, N.; Okamoto, Y.; Umakoshi, H. Insight into the Exosomal Membrane: From Viewpoints of Membrane Fluidity and Polarity. *Langmuir* **2021**, *37* (38), 11195–11202. https://doi.org/10.1021/acs.langmuir.1c00687.

(11) Nakayama, S.; Suga, K.; Kamata, T.; Watanabe, K.; Namigata, H.; Welling, T. A. J.; Nagao, D. Bio-Inspired Polydiacetylene Vesicles for Controlling Stimulus Sensitivity. *Macromol. React. Eng.* **2025**, *19* (1), 2400016. https://doi.org/10.1002/MREN.202400016.

(12) Suga, K.; Murakami, M.; Nakayama, S.; Watanabe, K.; Yamada, S.; Tsuji, T.; Nagao, D. Surface Characteristics of Antibacterial Polystyrene Nanoparticles Synthesized Using Cationic Initiator and Comonomers. *ACS Appl. Bio Mater.* **2022**. https://doi.org/10.1021/acsabm.2c00046.

(13) Nagasawa, A.; Fujishima, K.; Suga, K.; Watanabe, K.; Nagao, D. Quantitative Characterization of Localized Hydrophobicity Surrounding Polymer Particle Surfaces for Aqueous Antibacterial Colloids. *Langmuir* **2025**, *41* (1), 654–662. https://doi.org/10.1021/ACS.LANGMUIR.4C03896.

(14) Watanabe, N.; Goto, Y.; Suga, K.; Nyholm, T. K. M.; Slotte, J. P.; Umakoshi, H. Solvatochromic Modeling of Laurdan for Multiple Polarity Analysis of Dihydrosphingomyelin Bilayer. *Biophys. J.* **2019**, *116* (5), 874–883. https://doi.org/10.1016/j.bpj.2019.01.030.

(15) Pathak, P.; London, E. Measurement of Lipid Nanodomain (Raft) Formation and Size in Sphingomyelin/POPC/Cholesterol Vesicles Shows TX-100 and Transmembrane Helices Increase Domain Size by Coalescing Preexisting Nanodomains but Do Not Induce Domain Formation. *Biophys. J.* **2011**, *101* (10), 2417–2425. https://doi.org/10.1016/j.bpj.2011.08.059.

(16) Et-Thakafy, O.; Delorme, N.; Gaillard, C.; Mériadec, C.; Artzner, F.; Lopez, C.; Guyomarch, F. Mechanical Properties of Membranes Composed of Gel-Phaseor Fluid-Phase Phospholipids Probed on Liposomes by Atomic Force Spectroscopy. *Langmuir* **2017**, *33* (21), 5117–5126. https://doi.org/10.1021/ACS.LANGMUIR.7B00363.

(17) Drolle, E.; Kučerka, N.; Hoopes, M. I.; Choi, Y.; Katsaras, J.; Karttunen, M.; Leonenko, Z. Effect of Melatonin and Cholesterol on the Structure of DOPC and DPPC Membranes. *Biochim. Biophys. Acta - Biomembr.* **2013**, *1828* (9), 2247–2254. https://doi.org/10.1016/J.BBAMEM.2013.05.015.

(18) Belička, M.; Gerelli, Y.; Kučerka, N.; Fragneto, G. The Component Group Structure of DPPC Bilayers Obtained by Specular Neutron Reflectometry. *Soft Matter* **2015**, *11* (31), 6275–6283. https://doi.org/10.1039/C5SM00274E.
